# *Tbx1* and *Foxi3* genetically interact in the pharyngeal pouch endoderm in a mouse model for 22q11.2 deletion syndrome

**DOI:** 10.1371/journal.pgen.1008301

**Published:** 2019-08-14

**Authors:** Erica Hasten, Bernice E. Morrow

**Affiliations:** Department of Genetics, Albert Einstein College of Medicine, Bronx, New York, United States of America; Stanford University School of Medicine, UNITED STATES

## Abstract

We investigated whether *Tbx1*, the gene for 22q11.2 deletion syndrome (22q11.2DS) and *Foxi3*, both required for segmentation of the pharyngeal apparatus (PA) to individual arches, genetically interact. We found that all *Tbx1*^*+/-*^*;Foxi3*^*+/-*^ double heterozygous mouse embryos had thymus and parathyroid gland defects, similar to those in 22q11.2DS patients. We then examined *Tbx1* and *Foxi3* heterozygous, null as well as conditional *Tbx1*^*Cre*^ and *Sox17*^*2A-iCre/+*^ null mutant embryos. While *Tbx1*^*Cre/+*^*;Foxi3*^*f/f*^ embryos had absent thymus and parathyroid glands, *Foxi3*^*-/-*^ and *Sox17*^*2A-iCre/+*^*;Foxi3*^*f/f*^ endoderm conditional mutant embryos had in addition, interrupted aortic arch type B and retroesophageal origin of the right subclavian artery, which are all features of 22q11.2DS. *Tbx1*^*Cre/+*^*;Foxi3*^*f/f*^ embryos had failed invagination of the third pharyngeal pouch with greatly reduced *Gcm2* and *Foxn1* expression, thereby explaining the absence of thymus and parathyroid glands. Immunofluorescence on tissue sections with E-cadherin and ZO-1 antibodies in wildtype mouse embryos at E8.5-E10.5, revealed that multilayers of epithelial cells form where cells are invaginating as a normal process. We noted that excessive multilayers formed in *Foxi3*^*-/-*^, *Sox17*^*2A-iCre/+*^*;Foxi3*^*f/f*^ as well as *Tbx1* null mutant embryos where invagination should have occurred. Several genes expressed in the PA epithelia were downregulated in both *Tbx1* and *Foxi3* null mutant embryos including Notch pathway genes *Jag1*, *Hes1*, and *Hey1*, suggesting that they may, along with other genes, act downstream to explain the observed genetic interaction. We found Alcam and Fibronectin extracellular matrix proteins were reduced in expression in *Foxi3* null but not *Tbx1* null embryos, suggesting that some, but not all of the downstream mechanisms are shared.

## Author summary

The mechanisms required for segmentation of the pharyngeal apparatus (PA) to individual arches are not precisely delineated in mammalian species. Using mouse models, we found that two transcription factor genes, *Tbx1*, the gene for 22q11.2 deletion syndrome and *Foxi3*, genetically interact in the third pharyngeal pouch endoderm during thymus and parathyroid gland development. When examining wildtype embryos, we found that each arch is surrounded by epithelial cells derived from the endoderm and ectoderm that undergo dynamic processes during PA segmentation. Invagination co-occurs with formation of multilayers of epithelia that become juxtaposed. The cells are then reorganized to form a dual-layer of tightly intercalated cells at the mature pouch-cleft junction. In *Tbx1* and *Foxi3* null mutant embryos, these processes are disrupted. Further, the endoderm cells form extensive multilayers in the region where cells should normally invaginate. When examining genes that may act downstream of *Tbx1* and *Foxi3* we found several including Notch pathway genes *Jag1*, *Hes1*, *and Hey1* are downregulated in both mutant embryos. Together, we show that *Tbx1* and *Foxi3* are important for regulating PA segmentation through cellular and genetic mechanisms that may be critical in 22q11.2 deletion syndrome patients.

## Introduction

The pharyngeal apparatus (PA) is an evolutionarily conserved structure that forms early in vertebrate embryos. The PA develops as a series of bulges, termed arches, found on the lateral surface of the head region of the embryo. During mammalian development, five pairs of pharyngeal arches numbered PA1, PA2, PA3, PA4, and PA6 (the fifth PA is transient) form subsequently, over time, from the rostral to caudal part of the head region of the embryo [1]. The process involved in the formation of each arch is referred to as pharyngeal segmentation. Each arch contributes to different craniofacial muscles, nerves and skeletal structures. PA1 contributes to the skull, incus and malleus of the middle ear, jaw, nerves and muscles of mastication. PA2, contributes to the skull, stapes in the middle ear, facial muscles, jaw, and upper neck skeletal structures. In addition to skeletal structures, muscles and nerves, PA3 is required to form the thymus and parathyroid glands. In mouse embryos, the thymus and parathyroid glands are derived only from PA3, but in humans the inferior parathyroid gland is derived from PA3 whereas PA4 contributes to the superior parathyroid gland. PA4 and PA6 contribute to the aortic arch and arterial branches [2, 3].

Each pharyngeal arch is surrounded by endoderm and ectoderm derived epithelial cells forming pharyngeal pouches and clefts, respectively. Mesoderm and neural crest derived mesenchyme cells occupy the center of each arch [1, 4]. The epithelia are needed to invaginate and promote segmentation to individual arches. The pharyngeal endoderm (PE), which is the focus of this study, receives and sends signals from the mesenchyme to initiate morphogenesis and invaginate [5]. Once each arch segments, proper patterning is also required to form derivative structures. The PE sends distinct signals in each arch to promote normal patterning [6–9]. Abnormal PA segmentation or patterning during development will cause defects within the later structures derived from the PA and leads to human birth defect disorders.

One particular gene important for PA segmentation is *Tbx1*, encoding a T-box transcription factor implicated in 22q11.2 deletion syndrome (DiGeorge syndrome [MIM# 188400]; velo-cardio-facial syndrome [MIM# 192430]). *Tbx1*^*-/-*^ homozygous null mutant mouse embryos die at birth with hypoplastic and intermittent missing craniofacial muscles [10], cleft palate, absent thymus and parathyroid glands, as well as a persistent truncus arteriosus (PTA) with a ventricular septal defect (VSD) [11–13]. The first arch forms in *Tbx1*^*-/-*^ embryos but the distal PA fails to become segmented, thereby explaining, in part, why the PA derived structures are malformed [11–13]. *Tbx1* is expressed in the mesoderm of the head region in early mouse embryos and then throughout the endoderm, mesoderm, and distal ectoderm of the PA, while each arch forms, and becomes reduced at mouse embryonic day (E)10.5 [12, 14, 15]. Tissue specific inactivation of *Tbx1* has been performed using the Cre-loxP system [4, 16–19]. It was found that *Tbx1* is required in all three tissues for development of the derivative organs affected in the null mutant embryos [17, 19–21]. Since the PE is critically important for segmentation of the distal PA [22, 23], it is important to understand the genes and processes that might act downstream.

Another gene that has been shown to be important for normal PA segmentation is *Foxi3*, which encodes a Forkhead box (Fox) transcription factor. *Foxi3* is expressed in the ectoderm in the head region early in embryonic development and is then expressed in the epithelia of the PA from around the same stages as when *Tbx1* is expressed [24]. *Foxi3* is also important for epithelial cell differentiation within the epidermis [25]. Heterozygous mutations were discovered in *Foxi3* in several hairless dog breeds with hair follicle and teeth defects [26]. Global *Foxi3*^-/-^ null mutant mouse embryos fail to form endodermal pouches and this results in failed PA segmentation leading to severe defects in the skull, jaw, and ears [27–29]. It has been shown that *Foxi3* may have a cell non-autonomous effect on craniofacial neural crest cell survival because these cells undergo apoptosis in the mutant embryos at E10.0 [27].

In this study, we tested whether there is a genetic interaction between *Tbx1* and *Foxi3* during mouse embryonic development. We discovered that these two factors interact at minimum, in the third pharyngeal pouch endoderm, needed to form the thymus and parathyroid glands. Further, we found that inactivation of *Foxi3* results in cardiovascular anomalies. We characterized the process of pharyngeal segmentation and found that global inactivation of *Tbx1* and *Foxi3* both result in failure of the epithelia to properly invaginate along with an expansion of multilayers of PE cells leading to failed segmentation of the distal PA. We identified some shared downstream genes that were reduced in expression in either null mutant and suggest that they may share some similar molecular mechanisms.

## Results

### *Tbx1* acts upstream of *Foxi3* in embryogenesis

**Fig 1:**
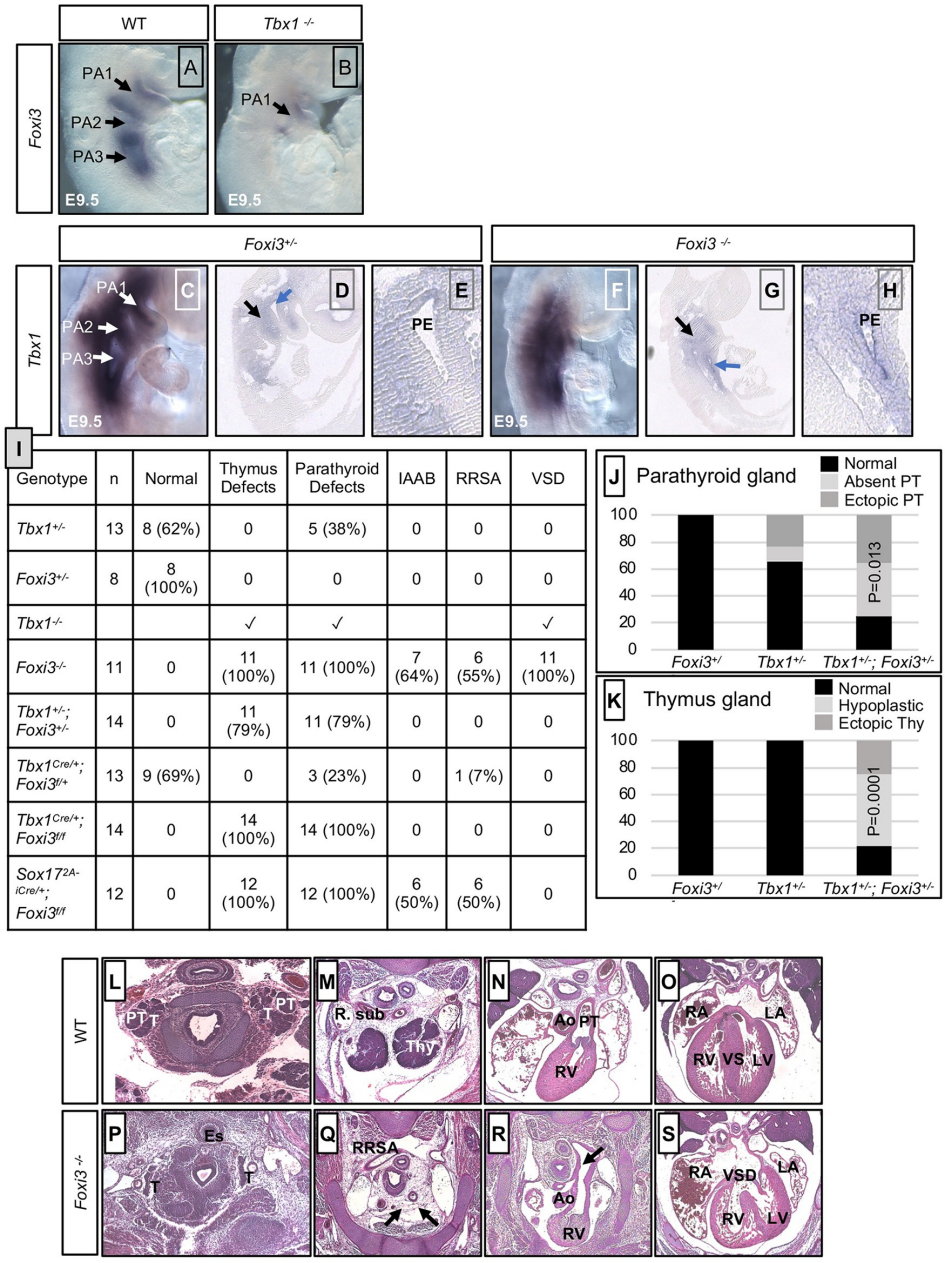
*Tbx1* and *Foxi3* genetically interact for thymus and parathyroid gland development. (A-B) Whole mount *in situ* hybridization (WMISH) using an antisense *Foxi3* probe on wildtype (WT; A) and *Tbx1*^*-/-*^ mouse embryos at E9.5 (B); n = 3 for each genotype. Individual pharyngeal arches, PA1, PA2 and PA3 are indicated and the arrows point to the corresponding arch. (C-H) WMISH using an antisense *Tbx1* probe on whole mount and sagittal sections of control (*Foxi3*^*+/-*^; C-E) and *Foxi3*^*-/-*^ (F-H) mouse embryos at E9.5 (n = 3 for each genotype). Black arrow indicates the core mesoderm and blue arrow indicates the endoderm. Zoomed in image of the pharyngeal pouch (E, H) next to the blue arrow (D, G), to demonstrate that *Tbx1* is still expressed in the control and mutant embryos, respectively. Pharyngeal endoderm is indicated as PE. (I) Table summarizing defects found in each embryo with the genotype listed in the first column on the left. The second column indicates the number of embryos. The rest of the columns indicate the number and percent (in parentheses) with various defects as determined by histological analysis at E15.5. Absent thymus and parathyroid glands were observed in all *Tbx1*^*-/-*^, *Foxi3*^*-/-*^ and *Sox17*^*2A-iCre/+*^*;Foxi3*^*f/f*^ embryos. Abbreviations: interrupted aortic arch type B (IAAB), retro-esophageal right subclavian artery (RRSA) and ventricular septal defect (VSD). (J-K) Bar graphs summarizing parathyroid (J) and thymus (K) defects found in *Tbx1*^*+/-*^, *Foxi3*^*+/-*^, and *Tbx1*^*+/-*^*;Foxi3*^*+/-*^ embryos versus controls. Fisher’s exact two tailed test was used to determine significance between defects observed in *Tbx1*^*+/-*^ and *Tbx1*^*+/-*^*;Foxi3*^*+/-*^ embryos. Defects observed in *Tbx1*^*+/-*^*;Foxi3*^*+/-*^ embryos include ectopic or absent parathyroid glands as well as ectopic and hypoplastic or absent thymus glands. (L-S) Histological sections of control (WT) embryos (L-O) and *Foxi3*^*-/-*^ embryos (P-S) at E15.5. *Foxi3*^*-/-*^ embryo with absent thymus glands and RRSA is shown (Q); IAAB is also present (R) as well as a VSD (S). Abbreviations: right subclavian artery (R. sub), thymus (Thy), aorta (Ao), pulmonary trunk (PT), right atrium (RA), left atrium (LA), right ventricle (RV), left ventricle (LV), ventricular septum (VS). More examples of defects that occurred in other mutant embryos are shown in S1 and S4 Figs.

Since loss of *Foxi3* or *Tbx1* disrupt the segmentation of the PA, we tested whether *Foxi3* might act upstream or downstream of *Tbx1*. *Foxi3* is normally expressed throughout the epithelia of the PA, while *Tbx1* is more broadly expressed (Fig 1A and 1C). Whole mount *in situ* hybridization (WMISH) using a *Foxi3* antisense mRNA probe on *Tbx1*^*-/-*^ mouse embryos and wildtype (WT) littermate controls at E9.5, revealed that *Foxi3* expression was reduced in PA1 and absent in the unsegmented distal pharyngeal apparatus in *Tbx1* null mutant embryos (Fig 1A and 1B). To determine if *Tbx1* expression is affected in *Foxi3*^*-/-*^ embryos, WMISH followed by tissue sectioning using a *Tbx1* probe on *Foxi3*^*+/-*^ control littermate and *Foxi3*^*-/-*^ mouse embryos was performed and we found that the *Tbx1* expression pattern was maintained in the pharyngeal mesoderm and endoderm despite the lack of segmentation of the distal PA (Fig 1C–1H). This indicates that either *Foxi3* acts downstream of *Tbx1* in the same genetic pathway and/or that the cells expressing *Foxi3* were lost in the *Tbx1* null mutant embryos.

### Inactivation of *Foxi3* leads to thymus, parathyroid and aortic arch defects

It has been previously shown that *Foxi3*^-/-^ embryos have absent jaw bones, abnormal mandible, deformed maxilla bones, absent jugal (bony arch of zygoma, cheek bone) and smaller palatines, misshapen Meckel’s cartilage, and absent ears [27, 29]. At E9.5, segmentation of the PA to individual arches did not occur in *Foxi3*^*-/-*^ embryos (Fig 1F and 1G), which is consistent with previous findings [27]. There is little known about its role in formation of later embryonic structures from the distal PA derived from PA3-6. We found that at E15.5, *Foxi3*^-/-^ embryos had absent thymus and parathyroid glands (100%; n = 11), interrupted aortic arch type B (IAAB, 63%; n = 7), ventricular septal defect (VSD; 100%; n = 11) and retro-esophageal right subclavian artery (RRSA, 55%; n = 6) as listed in Fig 1I and shown in Fig 1L–1S. Some had both a RRSA and IAAB (18%; n = 2), while the remaining had either RRSA or IAAB (81%; n = 9; Fig 1I and 1P–1S).

### *Tbx1* and *Foxi3* genetically interact in the pharyngeal apparatus

**Fig 2:**
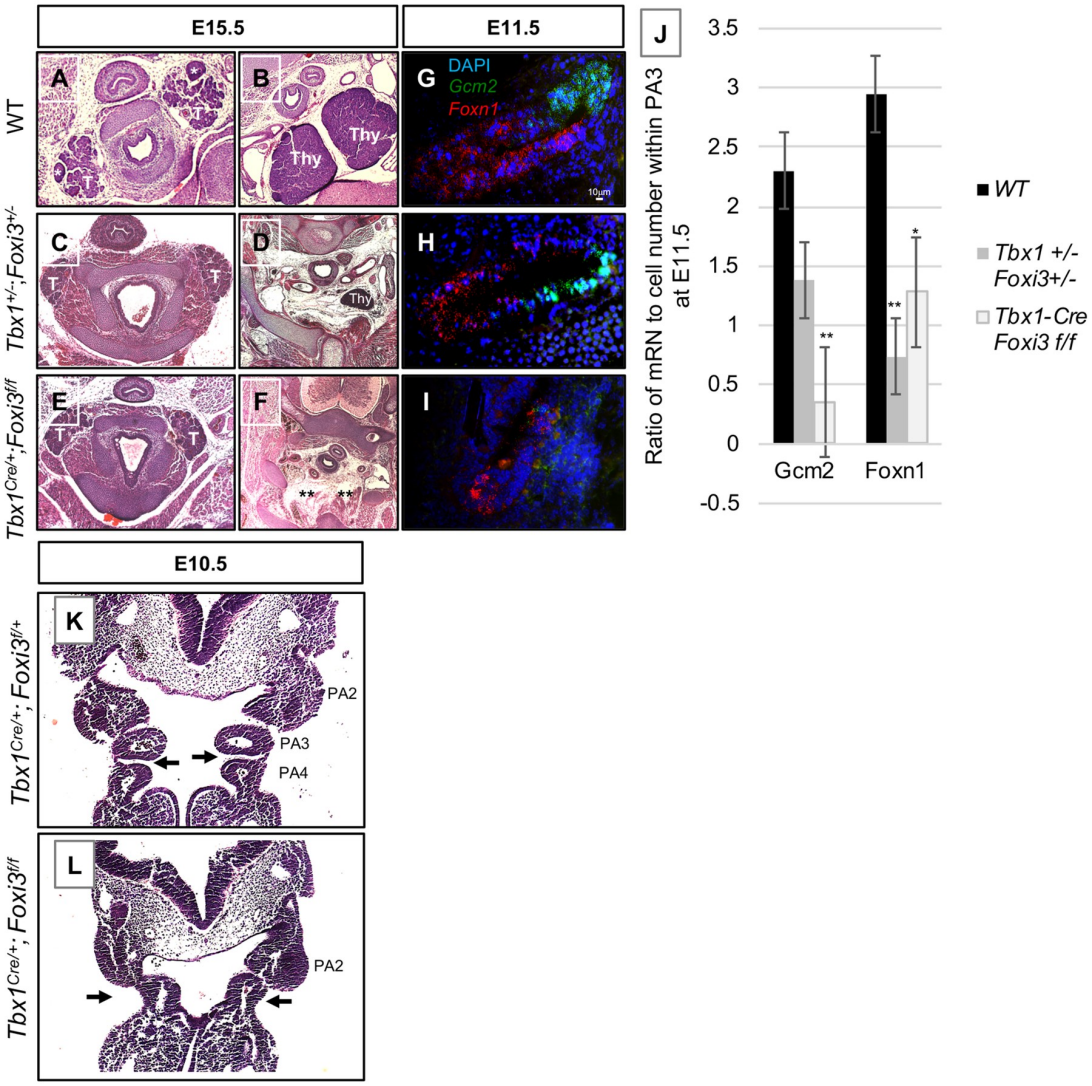
Thymus and parathyroid gland defects in *Tbx1*^*+/-*^*;Foxi3*^*+/-*^ and *Tbx1*^*Cre/+*^*;Foxi3*^*f/f*^ embryos. (A-F) Transverse histology sections stained with H&E of WT (A-B), *Tbx1*^*+/-*^*;Foxi3*^*+/-*^ (C-D) and *Tbx1*^*Cre/+*^*;Foxi3*^*f/f*^ (E-F) embryos at E15.5. Abbreviations: thyroid (T) and thymus (Thy). Asterisks in A indicate parathyroid glands. Asterisks in F indicate absent thymus glands. Numbers include: WT, n = 16; *Tbx1*^*+/-*^*;Foxi3*^*+/-*^, n = 13; *Tbx1*^*Cre/+*^*;Foxi3*^*f/f*^, n = 14. Normal parathyroid glands are located adjacent to thyroid glands (A) as compared to when they are not present adjacent to the thyroid glands in mutant embryos (C, E). Thymus glands are located at the branchpoint between the innominate and right carotid artery (B) but are more rostrally located and smaller (D) or absent (E) in mutant embryos. (G-I) RNAscope *in situ* hybridization with mRNA probes for *Gcm2* (green) and *Foxn1* (red) probes on sagittal sections in WT (G), *Tbx1*^*+/-*^*;Foxi3*^*+/-*^ (H) and *Tbx1*^*Cre/+*^*;Foxi3*^*f/f*^ (I) embryos at E11.5. *Gcm2* marks parathyroid gland precursor cells and *Foxn1* marks thymus gland precursor cells; n = 3 for each genotype. (J) Quantification of RNAscope experiments. Number of mRNA signal dots was quantified in proportion to number of cells present in each tissue section. P-values were determined using the t-test. Two stars indicate a P-value <0.005 and one star indicates a P-value <0.05. (K-L) H&E coronal histology sections of *Tbx1*^*Cre/+*^*;Foxi3*^*f/+*^ (K), and *Tbx1*^*Cre/+*^*;Foxi3*^*f/f*^ (L) embryos at E10.5. Arrows indicate the third pharyngeal pouch and morphology defects. Third pouch is absent in L. *Tbx1*^*Cre/+*^*;Foxi3*^*f/+*^, n = 6 and *Tbx1*^*Cre/+*^*;Foxi3*^*f/f*^, n = 8.

Based upon the similarities in the distal PA derived defects, and that *Foxi3* was reduced in expression in *Tbx1*^*-/-*^ embryos, we tested whether there could be a genetic interaction between the two genes. We first tested whether single heterozygous *Foxi3*^*+/-*^ [27] or *Tbx1*^*+/-*^ [11] embryos had defects. At E15.5, *Fox3*^*+/-*^ embryos were normal (n = 8) and *Tbx1*^*+/-*^ embryos had a normal thymus gland and had ectopic or absent parathyroid glands in 38% of the embryos (n = 5; Fig 1I–1K; S1A–S1H Fig). Normally, the parathyroid glands should be found adjacent to the thyroid glands, where the two carotid arteries are present nearby in the same section. When ectopic in *Tbx1*^*+/-*^ or *Tbx1*^*+/-*^*;Foxi3*^*+/-*^ embryos, parathyroid glands were found in a more caudal position in the embryo that is separate from the thyroid glands (Fig 2C; S1C and S1 Fig) versus controls (Fig 2A; S1A Fig). When ectopic in *Tbx1*^*+/-*^*;Foxi3*^*+/-*^ embryos, thymus glands were more rostrally located than normal and were present at the same level of the embryos as the carotid arteries (Fig 2D; S1F Fig), as compared to control embryos, where the thymus glands were located at the branchpoint between the innominate and right carotid artery (Fig 2B; S1B and S1D Fig). Hypoplastic thymus glands were smaller in size than normal glands (Fig 2D; S1F Fig). At E15.5, all double heterozygous *Tbx1*^*+/-*^*;Foxi3*^*+/-*^ embryos had either a hypoplastic and/or ectopic thymus and parathyroid glands (n = 14) and this increase is statistically significant (Fig 1J and 1K). More than half of double heterozygous embryos had both a hypoplastic thymus and ectopic parathyroid glands in comparison to WT controls (57%; n = 8/14; Figs 1J, 1K and 2A–2D; S1A–S1H Fig).

Expression of *Gcm2* (glial cells missing homolog 2) and *Foxn1* (Forkhead box protein N1) mark the parathyroid-fated and thymus-fated domains in the pharyngeal endoderm of PA3, respectively [30–32]. We performed RNAscope *in situ* hybridization on tissue sections with probes for *Gcm2* and *Foxn1* at E11.5 when both of these genes are expressed. In *Tbx1*^*+/-*^*;Foxi3*^*+/-*^ embryos, expression of both markers was slightly reduced in intensity in PA3 in comparison to WT littermate controls (Fig 2G and 2H). When expression was quantified in comparison to WT embryos, both genes were reduced in expression in the PA3 derivative region in the double heterozygous embryos, but only reduction of *Foxn1* was statistically significant (Fig 2J). The presence of some expression of these two genes is consistent with the occurrence of milder thymus and parathyroid gland defects (hypoplastic thymus and/or ectopic parathyroid) in these embryos as compared to null mutant embryos (Fig 1I; S1A–S1H Fig; Fig 2A–2D). Histology sections of *Tbx1*^*+/-*^*;Foxi3*^*+/-*^ embryos at E10.5 were examined to see if there were defects in PA3. We noted a slightly narrowing of the space between PA3 and PA4 in the double heterozygous embryos, but no other malformations were detected (S1I–S1M Fig). Again, the phenotype at E15.5 is relatively mild in comparison to either null mutant, in which the organs were completely absent. To gain more insights into the basis of the observed defects, we examined where *Tbx1* and *Foxi3* are co-expressed.

### *Tbx1* and *Foxi3* are co-expressed in the pharyngeal pouches and clefts

**Fig 3:**
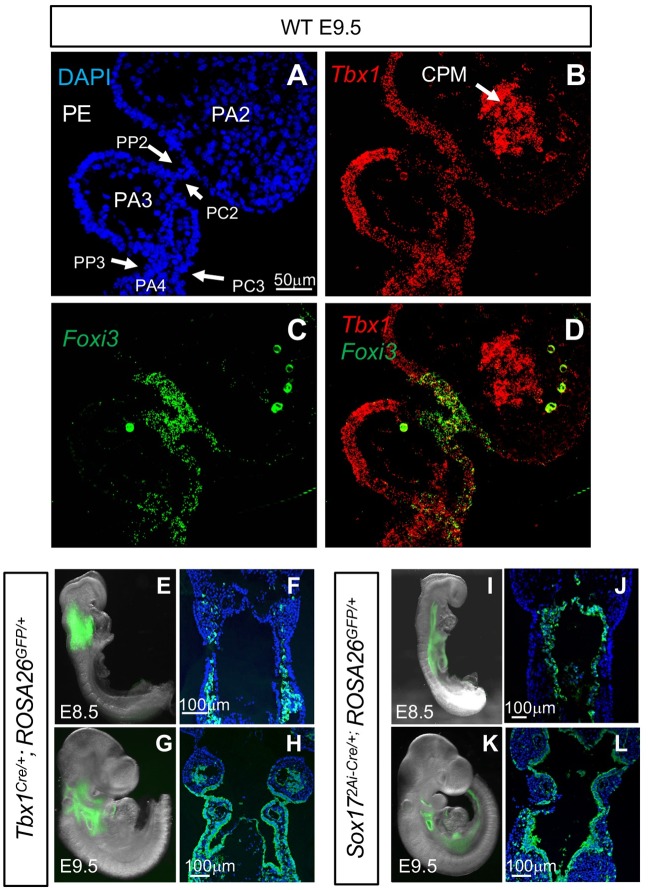
*Foxi3* and *Tbx1* expression in embryonic pharyngeal pouches and *Tbx1*^*Cre*^ and *Sox17*^*2A-iCre*^ lineages. (A-D) RNAscope *in situ* hybridization with mRNA probes for *Foxi3* and *Tbx1* on coronal sections from a WT embryo at E9.5. *Tbx1* expression is shown in red and *Foxi3* expression is shown in green. Abbreviations: Pharyngeal endoderm (PE); pharyngeal pouch 2 (PP2); pharyngeal cleft 2 (PC2); pharyngeal pouch 3 (PP3); pharyngeal cleft 3 (PC3); and cardiopharyngeal mesoderm (CPM). Total of n = 3 embryos. (E-H) Lineage tracing of *Tbx1*^*Cre/+*^*;Rosa26*^*GFPf/+*^ embryos. GFP expression is visible in whole mount embryos and the corresponding coronal sections at E8.5 (E-F) and at E9.5 (G-H); n = 3 for each genotype and stage. (I-L) Lineage tracing of *Sox17*^*2A-iCre/+*^;*Rosa26*^*GFPf/+*^ embryos. GFP expression is visible in whole mount embryos and the corresponding coronal sections at E8.5 (I-J) and E9.5 (K-L); n = 4 for each genotype and stage.

The PA develops from E8.5-E10.5, in which PA3 forms by E9.5. *In situ hybridization* using RNAscope probes was performed on coronal tissue sections from WT embryos to determine if there is overlap between *Foxi3* and *Tbx1* mRNA expression at E9.5 (Fig 3A–3D). There was strong expression of *Tbx1* in the pharyngeal endoderm and cardiopharyngeal mesoderm (Fig 3B), as has been previously reported [33, 34]. *Tbx1* was also expressed in the pharyngeal endoderm and ectoderm of the distal PA (Fig 3B) as has been previously reported [33]. *Foxi3* expression was localized exclusively to the pharyngeal pouches and clefts as published in the past [35]. Expression of *Foxi3* was particularly strong in the junction between the pharyngeal pouch and cleft that lies between PA2 and PA3 (Fig 3C). Expression of *Foxi3* was also detected where invagination of the epithelia is taking place to form the separation between PA3 and PA4 (Fig 3C). Co-expression of *Tbx1* and *Foxi3* in the same cells was detected in the second and third pharyngeal pouch and cleft and where invagination was taking place (Fig 3D). The third pharyngeal pouch is where co-expression of both genes occurred and this is the same region where the thymus and parathyroid glands will form. This provides supporting evidence that there could be a genetic interaction between the two genes. We then decided to inactivate both alleles of *Foxi3* using the *Tbx1*^*Cre*^ mouse line [36], where we would expect more obvious developmental defects at these early stages than when we inactivate one allele.

### Conditional inactivation of *Foxi3* results in defects in the distal pharyngeal apparatus

To establish the role of *Foxi3* within the *Tbx1* lineage to explain the basis of PA3 derived defects in double heterozygous embryos, we inactivated it using *Tbx1*^*Cre/+*^ knockin mice [36]. For this, *Tbx1*^*Cre/+*^ mice were crossed with a *Foxi3* floxed allele (*Foxi3*^*f/+*^) and the double heterozygous mice were crossed with *Foxi3*^*f/f*^ mice to inactivate both alleles of *Foxi3*. We also crossed the *Tbx1*^*Cre/+*^ mice with a *Rosa26*^*GFP/GFP*^ allele to detect the *Tbx1* lineage using the GFP reporter. GFP fluorescence was observed in the pharyngeal mesoderm at E8.5 (Fig 3E and 3F) and in both the pharyngeal mesoderm plus epithelia of the PA at E9.5 (Fig 3G and 3H). The *Tbx1*^*Cre/+*^ is a knock-in allele for *Tbx1* and it is heterozygous for *Tbx1* [36]. GFP fluorescence was not detected in the epithelia at E8.5. This was unexpected because *Tbx1* mRNA expression occurs in the epithelia at this stage [37]. Thus, there is a difference in timing of *Tbx1* expression and detection of GFP fluorescence, which marks recombination of loxP sites and translation of sufficient GFP to be visualized. This timing difference may explain why *Tbx1*^*Cre/+*^*;Foxi3*^*f/+*^ embryos at E15.5 did not exhibit a more severe phenotype than what occurred in *Tbx1*^*+/-*^ embryos, as compared to that in *Tbx1*^*+/-*^*;Foxi3*^*+/-*^ embryos (Fig 1I).

At E15.5, *Tbx1*^*Cre/+*^*;Foxi3*^*f/f*^ mutant embryos were compared to *Tbx1*^*Cre/+*^*;Foxi3*^*f/+*^ controls (n = 13) to determine if there were any PA derived defects (Fig 1I). Reduction of *Foxi3* expression in the pouches, but not the pharyngeal clefts, within the PA in *Tbx1*^*Cre/+*^*;Foxi3*^*f/f*^ embryos was observed in S2 Fig. The *Tbx1*^*Cre/+;*^*Foxi3*^*f/f*^ embryos had absent thymus and parathyroid glands (100% n = 14; Fig 2E and 2F), similar to what occurred in *Foxi3*^*-/-*^ embryos (Fig 1I). To determine if *Foxn1* (thymus) and *Gcm2* (parathyroid) expression [32] was affected, we performed RNAscope *in situ* hybridization with probes for these genes and found that the expression of both genes in the PA3 region were significantly reduced in conditional null versus wildtype controls (Fig 2G, 2I and 2J). We noted that there was no separation between PA3 and PA4 at stage E10.5 (Fig 2L) as compared to the presence of a separation in *Tbx1*^*Cre/+*^*;Foxi3*^*f/+*^ controls (Fig 2K). Reduction in expression of *Foxn1* and *Gcm2* as well as the presence of morphology defects at E10.5 might explain why the thymus and parathyroid glands did not form. Despite having absent thymus and parathyroid glands, these mutant embryos did not have cardiovascular or aortic arch defects (Fig 1I and S3A–S3F Fig). India ink injections confirmed presence of aortic arch arteries 3, 4 and 6, in WT and *Tbx1*^*Cre/+*^*;Foxi3*^*f/f*^ embryos (S3G and S3H Fig). While expression of *Foxi3* was significantly reduced as determined by WMISH (S3A–S3D Fig) in conditional mutant embryos, we also tested *Tbx1*^*Cre/+*^*;Foxi3*^*f/-*^ embryos to further inactivate *Foxi3* and found that the embryos lacked the thymus and parathyroid glands but similar to above, they had no intracardiac or aortic arch defects (n = 8; S3I–S3L Fig). Therefore, there is an interaction between *Tbx1* and *Foxi3* in the third pharyngeal pouch endoderm. We did not detect PA4 derived defects in the *Tbx1*^*Cre/+*^*;Foxi3*^*f/-*^ embryos at E15.5. There are two different possibilities to explain basis for the lack of aortic arch or branching anomalies derived from PA4. One possibility is that there is no genetic interaction in the fourth pharyngeal pouch. Another possibility could be due to timing of *Tbx1* gene expression and delayed timing of Cre activity using the *Tbx1*^*Cre*^ allele, within the PE (Fig 3E–3H), although its less likely an issue in *Tbx1*^*Cre/+*^*;Foxi3*^*f/-*^ embryos. We then next decided to inactivate *Foxi3* in the PE to understand its tissue specific function.

*Foxi3* is expressed in the epithelia in the PA (Figs 1A, 3C and 3D and [24]). To determine the role of *Foxi3* within the PE, we performed tissue specific inactivation of *Foxi3* using the *Sox17*^*2A-iCre/+*^ allele [38]. We first confirmed that the PE population (and endothelial cells) is marked by GFP expression using *Sox17*^*2A-iCre/+*^*;ROSA26*^*GFP/+*^ embryos (Fig 3I–3L). *Foxi3* is not expressed in endothelial cells. Recombination occurred prior to E8.5 as indicated by robust green fluorescence that was present at E8.5 (Fig 3I–3L). *Foxi3* expression was reduced in these conditional mutants as detected by WMISH (S2E–S2H Fig). We found that *Sox17*^*2A-iCre/+*^*;Foxi3*^*f/f*^ embryos had failed segmentation of the distal PA as compared to *Sox17*^*2A-iCre/+*^*;Foxi3*^*f/+*^ controls (S4A and S4C Fig). The PA was not as severely affected as in the *Foxi3*^*-/-*^ null mutant embryos (S4B Fig), as PA2 was present, albeit hypoplastic (S4C Fig). At E15.5, all *Sox17*^*2A-iCre/+*^*;Foxi3*^*f/f*^ embryos had absent thymus and parathyroid glands (100%; n = 12; Fig 1I). Cardiovascular defects occurred in 66% of embryos that included IAAB (50% n = 6), and/or RRSA (50% n = 6; Fig 1I and S4D–S4F Fig). As compared to *Foxi3*^*-/-*^ mutant embryos that always had an aortic arch defect plus a VSD, *Sox17*^*2A-iCre/+*^*;Foxi3*^*f/f*^ mutant embryos had an IAAB but had normal septation of the ventricles (Fig 1I). Several mutant embryos had both an RRSA and IAAB (33% n = 3), while 33% (n = 3) had just an RRSA and 33% (n = 3) had an IAAB, whereas a few had normal aortic arches and normal branching (33% n = 3; Fig 1I). India ink injections showed that the 4^th^ aortic arch artery was absent in *Sox17*^*2A-iCre/+*^*;Foxi3*^*f/f*^ embryos at E10.5, similar to what we observed in *Foxi3*^*-/-*^ embryos (S4G–S4L Fig). Absence of the 4^th^ aortic arch arteries would lead to the observed cardiovascular defects that occurred in the embryos. Based upon this data, *Foxi3* expression is required in the PE for classic 22q11.2DS phenotypes. Further, *Foxi3* in the ectoderm may have an additional role in the septation of the ventricles.

### Loss of *Foxi3* in the *Tbx1* expressing lineage disrupts segmentation between PA3-4

**Fig 4:**
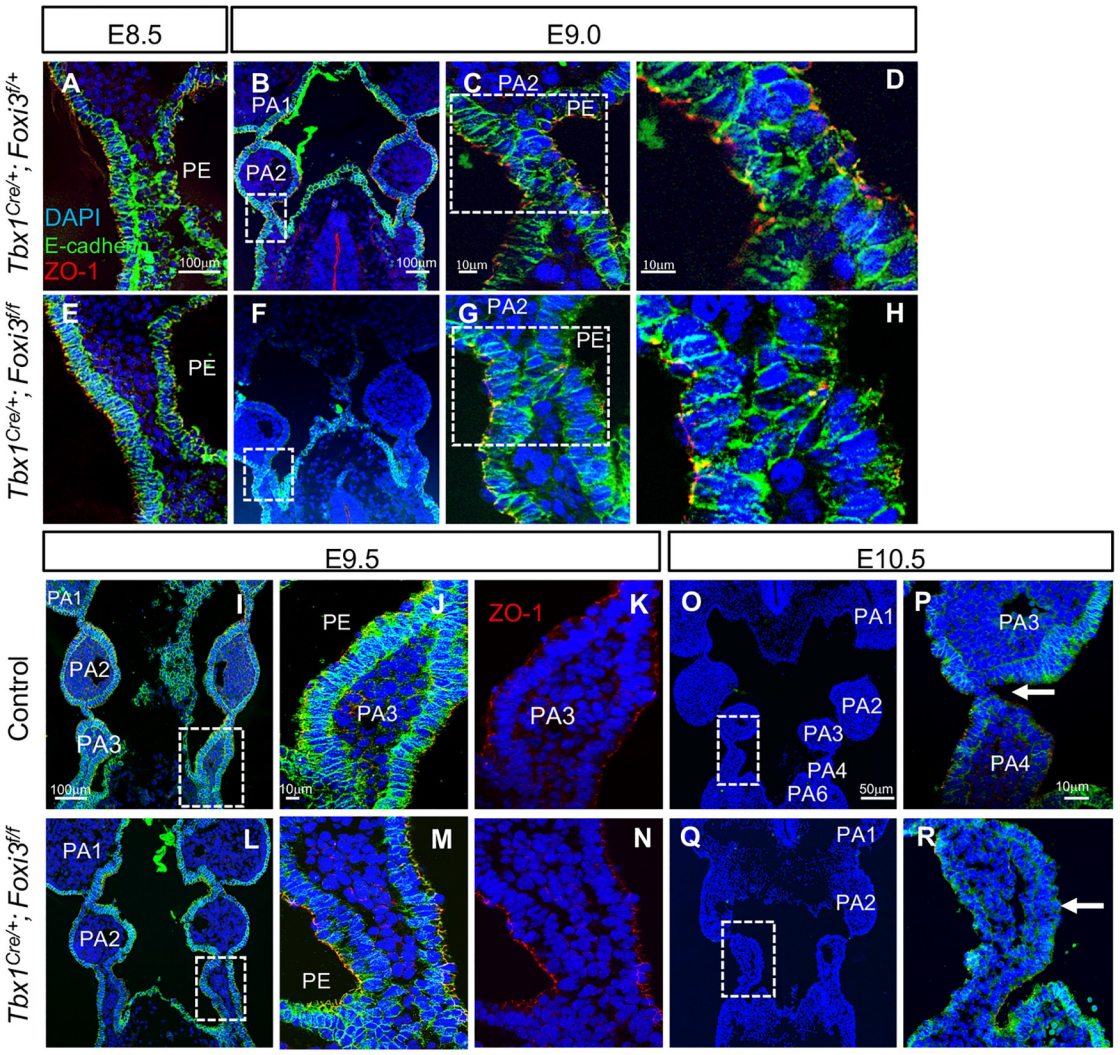
Inactivation of *Foxi3* within the *Tbx1*^*Cre/+*^ lineage disrupts PA3 morphogenesis. (A-R) DAPI (blue), E-cadherin (green), and ZO-1 (red) antibodies were used for immunofluorescence on coronal sections from embryos at E8.5-E9.0 (n = 3, each), E9.5 (n = 5) and E10.5 (n = 4) in *Tbx1*^*Cre/+*^*;Foxi3*^*f/+*^ (A-D, O-P) or WT (I-K) controls and in *Tbx1*^*Cre/+*^*;Foxi3*^*f/f*^ (E-H, L-N, Q-R) mutant embryos. White boxes indicate the location of the higher magnification images. White arrows in E10.5 images (P, R) indicate 3^rd^ pharyngeal pouch in control and absent 3^rd^ pharyngeal pouch in mutant embryos.

In the *Tbx1*^*Cre/+*^*;Foxi3*^*f/f*^ mutant embryos, only PA3 derivative structures were affected, being the thymus and parathyroid glands. As indicated above, the pouch and cleft between PA3 and PA4 did not form and was missing at E10.5, when segmentation was complete (Fig 2K and 2L). E-cadherin is a cell-cell adhesion protein forming adherens junctions that bind cells tightly to each other and it marks epithelial cells (Reviewed in [39]). ZO-1 (Zona occluden-1) forms permeable barriers in adherens junctions and is a marker for the presence of apical/basal polarity among epithelial cells, with expression specifically on the apical side of the cell facing a lumen [40]. Immunofluorescence using antibodies to E-cadherin and ZO-1 was performed on WT, *Tbx1*^*Cre/+*^*;Foxi3*^*f/+*^ and *Tbx1*^*Cre/+*^*;Foxi3*^*f/f*^ embryos at E9.5, to visualize the structure of the epithelial cell population. At E8.5 and E9.0 there was no noticeable difference between the mutant and control embryos (Fig 4A–4H). But, at E9.5, invagination of the partially stratified pharyngeal endoderm and ectoderm between PA3 and PA4 did not take place (Fig 4L–4N) as compared to controls (Fig 4I–4K). The PE of PA3 maintained its epithelial identity and cell polarity as marked by expression of E-cadherin (Fig 4I, 4J, 4L and 4M) and ZO-1 (Fig 4K and 4N). It is possible that instead of failure to invaginate, there was a delay. However, at E10.5, the third pouch and cleft were absent in the conditional mutant embryos (Fig 4Q and 4R) in comparison to controls (Fig 4O and 4P), so that there was no distinction between PA3 and PA4 as shown by H&E staining (Fig 2K and 2L). Thus, the genetic interaction between the two genes is at minimum, in PA3.

### Process of pharyngeal segmentation in wildtype and mutant embryos

**Fig 5:**
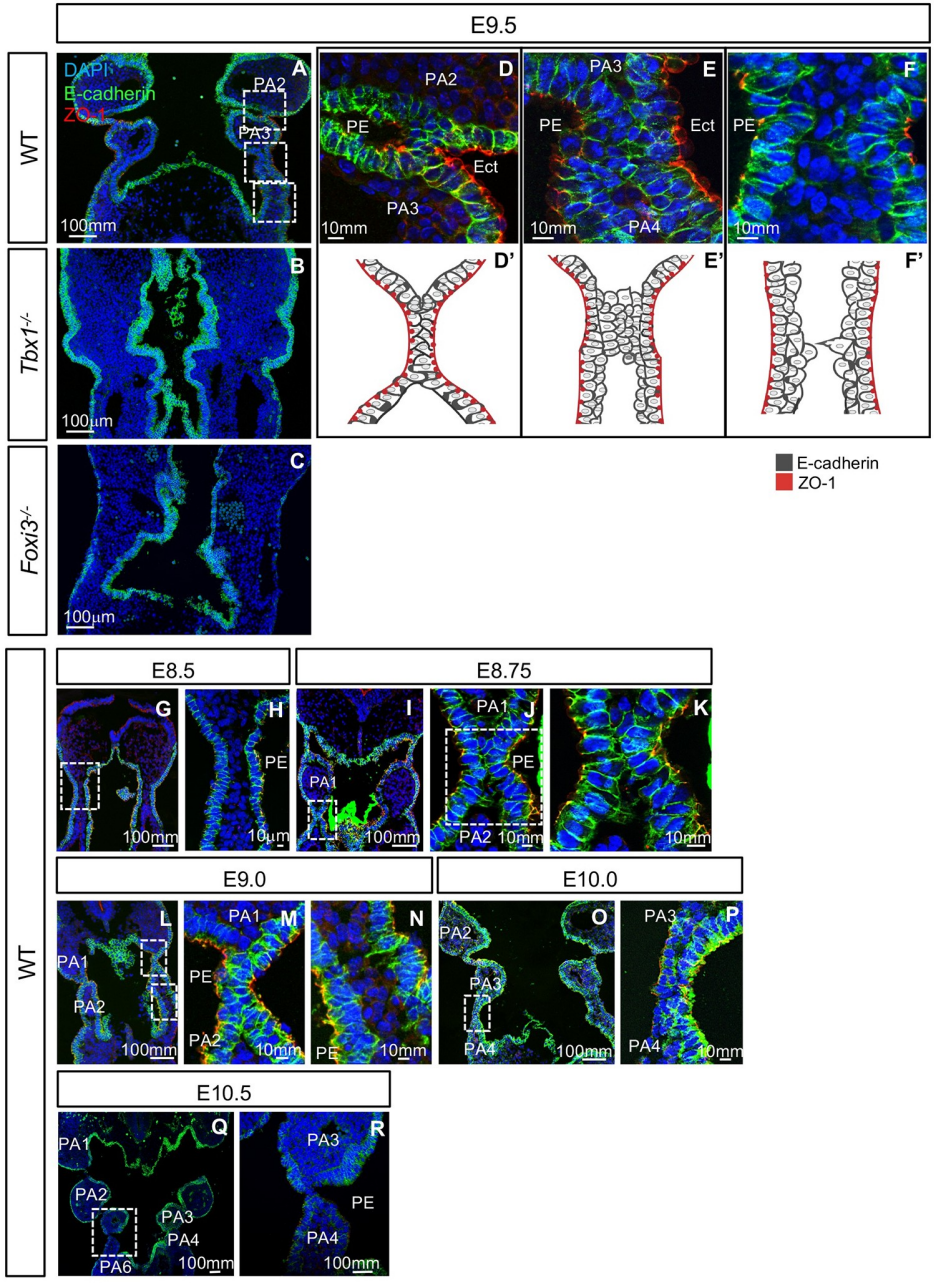
PA segmentation in wildtype, *Tbx1* and *Foxi3* mutant embryos. (A-F) To visualize epithelial cells within the PA, DAPI (blue), E-cadherin (green), and ZO-1 (red) antibodies were used for immunofluorescence on WT (A), *Tbx1*^*-/-*^ (B), and *Foxi3*^*-/-*^ (C) coronal sections at E9.5 (WT, n = 5; *Tbx1*^*-/-*^, n = 4 and *Foxi3*^*-/-*^, n = 5). White boxes (A) indicate where the region was magnified (D-F). (D) Second pouch and cleft where segmentation is complete and epithelial cells formed a dual intercalated layer. (E) Third pouch and clefts are forming at E9.5. The epithelium is stratified. (F) Location of future PA4-PA6, where invagination is initiated, as can be observed by cell projections from the epithelia. PE indicates the pharyngeal endoderm and Ect. indicates the ectoderm for each section. (D’-F’) Cartoon illustrating the segmentation process from images in D-F. (G-R). DAPI (blue), E-cadherin (green), and ZO-1 (red) antibodies were used for immunofluorescence to visualize epithelial cells within the PA in coronal sections from WT E8.5 (G-H), E8.75 (I-K), E9.0 (L-M), E10.0 (O-P), E10.5 (Q-R) embryos. White boxes indicate the location of higher magnification images. PE indicates pharyngeal endoderm; n = 4 for each stage.

Immunofluorescence using antibodies to E-cadherin and ZO-1 was performed to detect differences between WT, *Tbx1* and *Foxi3* null mutant embryos at E9.5. While individual arches were present in the PA in WT embryos, segmentation of the distal PA, PA2-6, did not occur in *Tbx1* or *Foxi3* null mutant embryos at this stage (Fig 5A–5C). In the *Tbx1*^*-/-*^ and *Foxi3*^*-/-*^ embryos, there was a multilayered stratified epithelium, possibly in areas where cells would begin to invaginate, which appeared thicker than in WT embryos (Fig 5A–5C).

To examine whether multilayers of epithelia are present as a normal part of epithelial cell dynamics, we carefully examined the PA segments in WT embryos at E9.5 (Fig 5A and 5D–5F). During normal segmentation of the distal PA, the endoderm and ectoderm invaginate toward each other to form a pouch and cleft, which becomes juxtaposed thereby providing a physical separation of the rostral and caudal arch. This process occurs dynamically in a rostral to caudal manner over time, such that the process of segmentation of different arches can be observed at one stage.

The junction of the pouch and cleft between PA2 and PA3 was completely formed by E9.5 and consisted of a tightly organized intercalated dual layer of cells, likely one layer of endoderm and one of ectoderm, in which ZO-1 is expressed at the outer face of each layer, on their apical surface (Fig 5D and 5D’). To determine the process of segmentation, we examined the region where the junction of the pouch and cleft between PA3 and PA4 was forming (Fig 5E and 5E’). We noted that there were multiple layers of a partially stratified epithelium where the pharyngeal epithelia became juxtaposed to each other (Fig 5E and 5E’). The outer cells expressed ZO-1 on their apical surface, but the inner cells did not (Fig 5E and 5F). It was as though a zippering process was beginning in the central region such that ZO-1 negative cells rostrally and caudally were being pushed out. More caudally, epithelial cells on either side of the mesenchyme appeared to be extending processes towards each other at the point where the cells were beginning to invaginate to form the next segment (Fig 5F and 5F’). Thus, the process of segmentation involves cell movement and repositioning as well as communication of the endoderm and ectoderm in order to form mature pouch-cleft junctions.

Additional stages of E8.5, E8.75, E9.0, E10.0 and E10.5 were examined to further characterize epithelial cell dynamics and ascertain whether there were fundamental differences at different stages in WT embryos (Fig 5G–5R). The first transition of pouch morphogenesis began at E8.5 when the endoderm and ectoderm initiate the process of invagination to eventually separate PA1 from PA2 (Fig 5G and 5H). We did not observe multilayers of epithelial cells at E8.5 (Fig 5G and 5H). As invagination was completing between PA1 and PA2, at E8.75 (Fig 5I), the pharyngeal endoderm and ectoderm, consisting of two to a few layers, became loosely juxtaposed (Fig 5J and 5K). This is similar to the pouch-cleft formation for PA3-4 at E9.5 (Fig 5E and 5E’). At E9.0 (Fig 5L), the junction between first pouch and cleft that separated PA1 and PA2 formed a tight intercalated dual cell layer (Fig 5M) that appears similar to that observed in Fig 5D and 5D’. At E9.0, the second pouch and cleft between PA2-PA3, consisting of a few layers of epithelium, became juxtaposed and intercalated (Fig 5N). At E9.0 and E9.5, there were two to three layers of endoderm cells in the caudal PA, as compared to less layers in the rostral PA, where the mature pouch-cleft junction occurred. At E10.0, the pouches and clefts formed a pouch-cleft junction that was almost mature in between PA3-4 (Fig 5O and 5P). At E10.5, PA formation was complete with the presence of dual layer mature pouch-cleft junctions between each arch (Fig 5Q and 5R).

### Global *Tbx1* inactivation results in excessive layers of endoderm cells

As indicated in Fig 5B, segmentation of the distal PA in *Tbx1*^*-/-*^ embryos did not occur and additional layers of epithelia were present at E9.5, possibly where the cells would begin to invaginate. However, proliferation assays at E8.5 and E9.5 revealed no significant changes in cell proliferation in null mutant embryos as compared to controls (S5A–S5G Fig) as has been previously reported at E9.5 [18]. ZO-1 expression was normal at E8.5 (S5H and S5J Fig) and E9.5 (S5I and S5K Fig), and the apical side of only the outer facing cells expressed ZO-1 (S5H–S5K Fig). The PA in *Tbx1*^*-/-*^ embryos was shorter in length and cell number quantification revealed that there were significantly more epithelial cells within the shortened PA at E9.5, but not at E8.5 (S5L Fig). This suggests that more cells were packed into a smaller PA at E9.5. We then performed endoderm specific inactivation to determine whether this process was cell type autonomous. Inactivation of *Tbx1* in the PE using the *Sox17*^*2A-iCre/+*^ allele results in a normal first and hypoplastic second arch, as compared to an absent second arch in global null mutant embryos. This is similar to the situation with endoderm inactivation of *Foxi3* (S4C Fig). Invagination of the PE did not take place and this resulted in failed segmentation of the distal arches [18], as we confirmed (S5M and S5N Fig). Similar to what was observed in the global *Tbx1* null mutant embryos, E-cadherin and ZO-1 expression was normal in the conditional mutant embryos as compared to the *Sox17*^*2A-iCre/+*^*;Tbx1*^*f/+*^ littermates at E9.5 and E10.5 (S5M and S5P Fig). As compared to the global null mutant, we did not observe excessive multilayers in the conditional mutant embryos at E9.5 (S5M and S5N Fig). We did observe multilayers of PE cells in the distal PA at E10.5 as compared to controls (S5O and S5P Fig), suggesting some differences between the conditional mutant embryos compared to null mutant embryos.

### Excessive multilayered epithelium occurs in the PA of *Foxi3* mutant embryos

**Fig 6:**
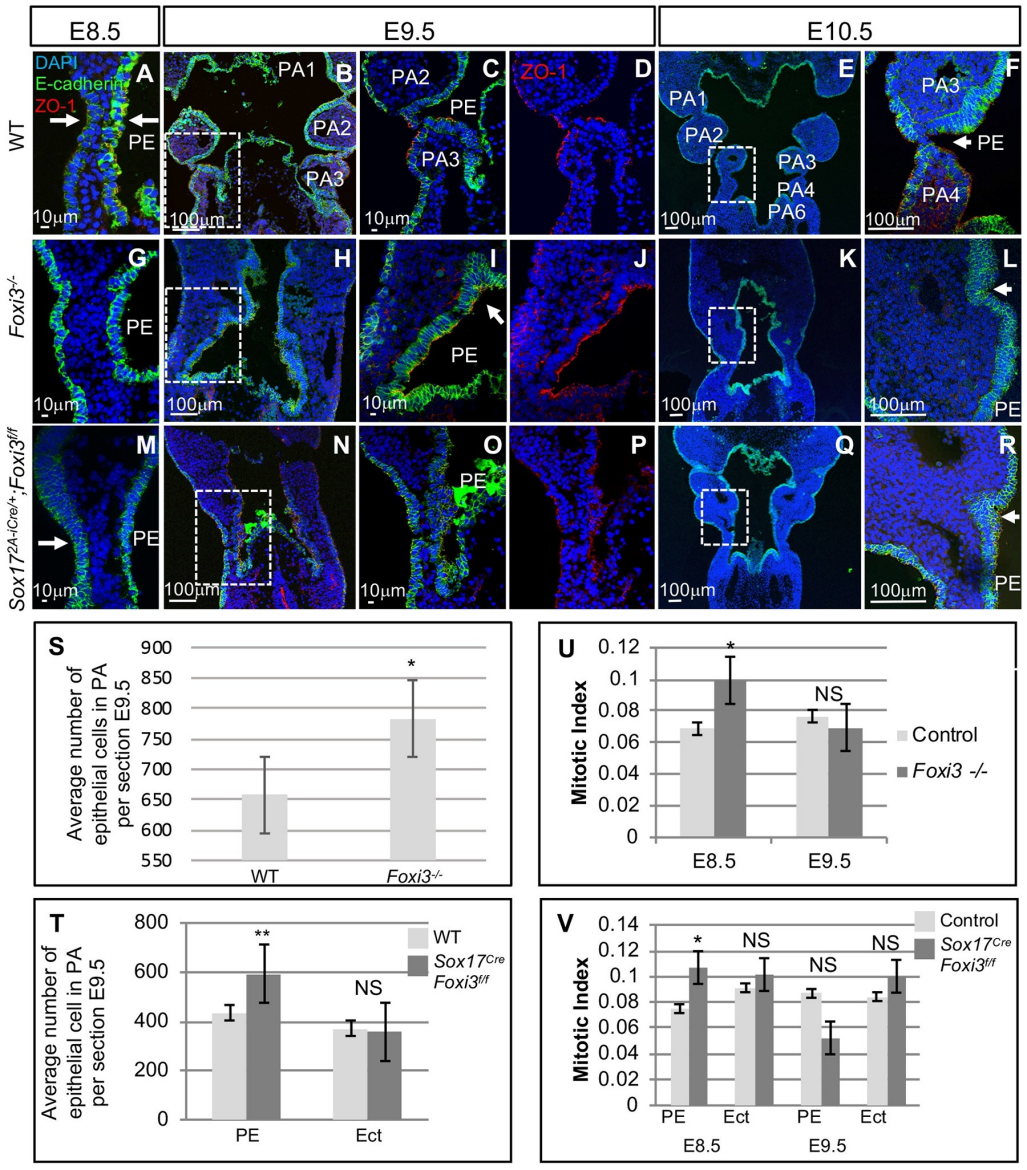
*Foxi3*^*-/-*^ and *Sox17-2A-iCre/+;Foxi3*^*f/f*^ mutant embryos have defects in PA segmentation. (A-R) DAPI (blue), E-cadherin (green), and ZO-1 (red) mark epithelial cells on coronal sections from WT (A-F), *Foxi3*^*-/-*^ (G-L) and *Sox17*^*2A-iCre/+*^*;Foxi3*^*f/f*^ (M-R) embryos from E8.5-E10.5 (n = 4, each). White boxes indicate the location of higher magnification images. White arrows indicate where invagination occurs in the PE and ectoderm. (S-T) Quantification of epithelial cells within the PA at E9.5 in WT controls and mutant embryos. Error bars represent standard error. One asterisk indicates P-value <0.05; two asterisks indicate P-value <0.005 (n = 4, each). (U-V) Quantification of proliferation assays includes counting E-cadherin positive epithelial cells within the PA and calculation of the mitotic index, which is the ratio of pH3 positive cells within the E-cadherin positive epithelial cells. Error bars represent standard error, and asterisks indicate P-value <0.05. Pharyngeal endoderm (PE) and ectoderm (Ect) were quantified separately in conditional mutant embryos. At E8.5 and E9.5 a total of n = 6 and n = 4, were examined, respectively, for control and mutant embryos. Controls include WT, *Foxi3*^*+/-*^, and *Sox17*^*2A-iCre/+*^*;Foxi3*^*f/+*^ littermates.

We next examined WT, *Foxi3*^*-/-*^ and *Sox17*^*2A-iCre/+*^*;Foxi3*^*f/f*^ embryos at E8.5-E10.5 to understand if the defects observed in *Foxi3* null mutant embryos occurred in a tissue specific manner (Fig 6). Invagination defects began at E8.5 in both *Foxi3*^*-/-*^ and *Sox17*^*2A-iCre/+*^*;Foxi3*^*f/f*^ embryos (Fig 6A, 6G and 6M). At E9.5, regions with excessive stratified multilayers of endoderm cells, especially at the points where the cells would be turning inwards to invaginate, were found in *Foxi3*^*-/-*^ (Fig 6H–6J) and *Sox17*^*2A-iCre/+*^*;Foxi3*^*f/f*^ embryos (Fig 6N–6P) versus WT controls (Fig 6B–6D). All endoderm cells in WT, *Foxi3*^*-/-*^ and *Sox17*^*2A-iCre/+*^*;Foxi3*^*f/f*^ embryos, expressed E-cadherin and the outermost cells expressed ZO-1 on the apical side, indicating that cells did not lose epithelial identity or polarity (Fig 6A–6R). At E9.5 and E10.5, the epithelial cells in *Foxi3*^*-/-*^ embryos began to invaginate but never advanced (Fig 6K and 6L) as compared to WT embryos (Fig 6E and 6F). At E10.5, the epithelia partially invaginated in the *Sox17*^*2A-iCre/+*^*;Foxi3*^*f/f*^ embryos, that seemed more complete for the ectoderm than endoderm (Fig 6Q and 6R). Overall, this data indicates a tissue autonomous role of *Foxi3* in the endoderm during segmentation of the PA.

### Cell proliferation is increased in *Foxi3* mutant embryos at E8.5 but not at E9.5

E-cadherin positive epithelial cells within the PA in WT versus *Foxi3*^*-/-*^ and WT embryos were quantified and there was a 45% (P-value <0.03) increase of epithelial cell number within the PA of *Foxi3* null mutants as compared to controls (S6 Fig) This may be correlated with the additional cell layers observed in the mutant embryos. There was a 40% increase in the number of endodermal cells (P-value <0.006), but no increase (P-value <0.4) was detected of ectoderm cells in the PA of *Sox17*^*2A-iCre/+*^*;Foxi3*^*f/f*^ mutant embryos versus controls, at E9.5 (Fig 6T). We then decided to test whether cell proliferation was increased at E8.5 and E9.5. For this, a pH3 antibody was used to mark proliferating cells on serial sections on mutant versus control embryos at E8.5 and E9.5 (Fig 6U and 6V; S6 Fig). When calculating the ratio between proliferating cells versus total E-cadherin positive cells, there was a significant increase at E8.5 in the endoderm of *Foxi3*^*-/-*^ and *Sox17*^*2A-iCre/+*^*;Foxi3*^*f/f*^ embryos versus controls (Fig 6U and 6V; S6 Fig). At E9.5 there was no difference in proliferation between mutant and control embryos (Fig 6U and 6V; S6 Fig). This indicates that increases of cell proliferation at E8.5 can partially explain why there is an increase of layers of epithelial cells within the PA at E9.5 but not at E10.5.

### Notch-pathway gene expression is reduced in *Foxi3* and *Tbx1* null embryos

**Fig 7:**
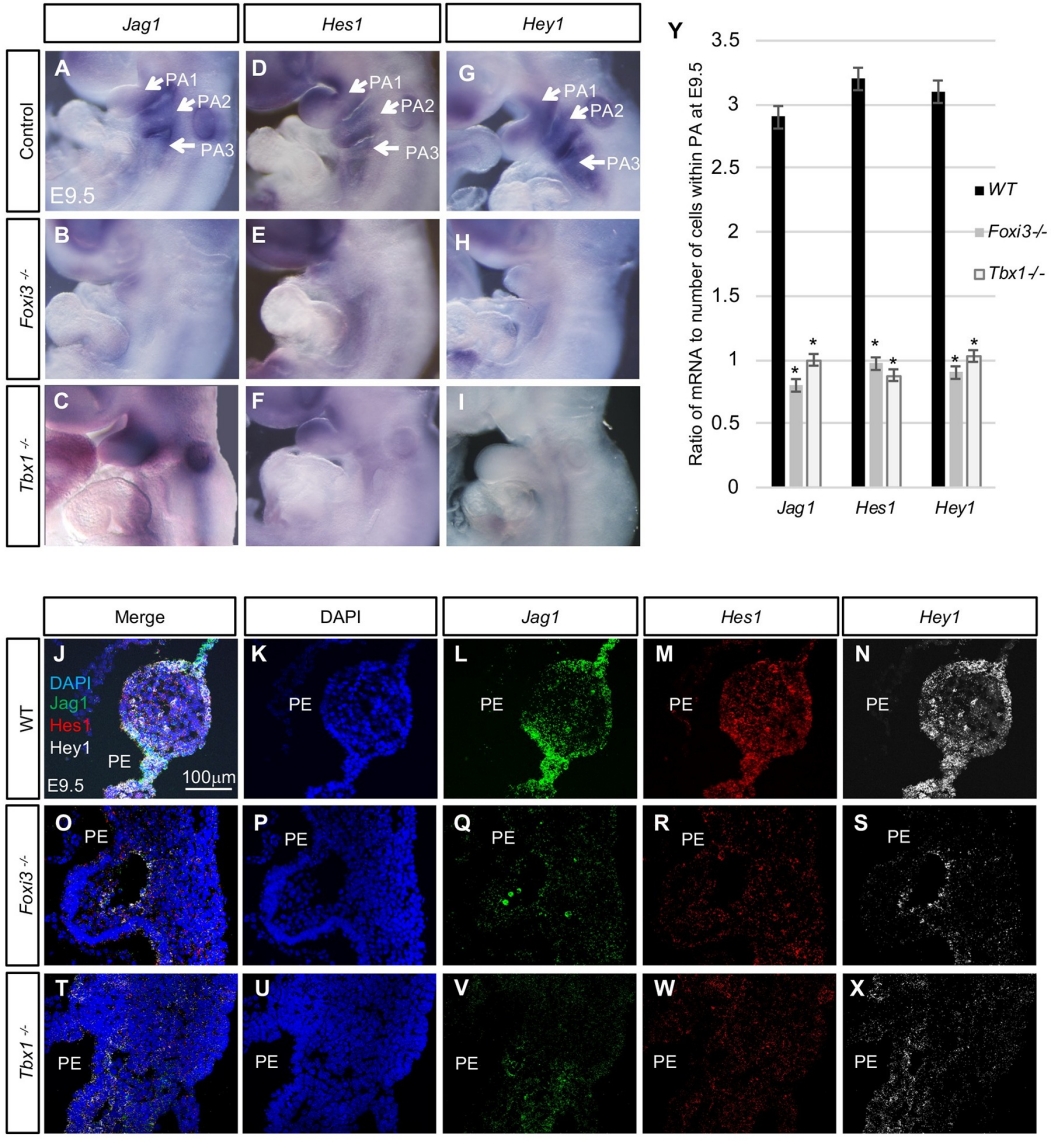
Expression of Notch pathway genes *Jag1*, *Hes1*, and *Hey1* were downregulated in *Foxi3* and *Tbx1* null mutant embryos. (A-I) WMISH with antisense *Jag1*, *Hes1*, and *Hey1* mRNA probes on whole mount embryos at E9.5. *Jag1* antisense probe on WT (A), *Foxi3*^*-/-*^ (B) and *Tbx1*^*-/-*^ mutant embryos (C); n = 3 of each genotype. *Hes1* probe on WT (D), *Foxi3*^*-/-*^ (E) and *Tbx1*^*-/-*^ (F) mutant embryos; n = 2–3 for each genotype. *Hey1* probe on WT (G), *Foxi3*^*-/-*^ (H) and *Tbx1*^*-/-*^ (I) mutant embryos; n = 2, each. (J-X) RNAscope *in situ* hybridization with mRNA probes for *Jag1*, *Hes1*, and *Hey1* on coronal sections of WT (J-N), *Foxi3*^*-/-*^ (O-S), and *Tbx1*^*-/-*^ (T-X) embryos at E9.5; n = 3, each genotype. Merged channels are shown (J, O, and T); DAPI is shown in blue color (K, P, and U); *Jag1* mRNA expression is in green (L, Q, and V); *Hes1* mRNA expression is in red (M, R, and W); and *Hey1* expression is in white (N, S and X). The sections correspond to the same rostral-caudal location within in each embryo. (Y) Quantification of RNAscope experiments on WT versus *Tbx1*^*-/-*^ and *Foxi3*^*-/-*^ embryos at E9.5. Nuclei and mRNA were quantified for each section and probe. The total number of mRNA signal dots was divided by the total number of cells for each replicate. Graph represents the average ratio. Asterisks indicate P-values < 0.05.

Notch signaling is critical for many aspects of embryonic development such as for skeletal development [41, 42] and cardiovascular development [21, 43, 44]. Notch signaling might have a possible role in thymus gland development [21, 45]. It has also been shown that Notch pathway genes, *Jagged1* (*Jag1*) and *Hes1* act downstream of *Tbx1* during embryogenesis [21, 46, 47]. We therefore wanted to determine if *Jag1*, *Hey1*, *and Hes1* may be regulated by *Tbx1* and *Foxi3* during PA formation. To test this, we performed WMISH and RNAscope experiments on WT, *Tbx1*^*-/-*^ and *Foxi3*^*-/-*^ mutant embryos at E9.5. By WMISH, *Jag1*, *Hes1* and *Hey1* expression in the pharyngeal pouch-cleft regions in WT embryos was reduced in both *Foxi3* and *Tbx1* null mutant embryos (Fig 7A–7I). Three-color RNAscope assays were performed on tissue sections from WT, *Tbx1*^*-/-*^ and *Foxi3*^*-/-*^ mutant embryos at E9.5, to examine expression level changes of *Jag1*, *Hes1* and *Hey1* (Fig 7J–7X). As in the WMISH experiments, *Jag1* expression was localized to the pouch-cleft junctions, *Hes1* was expressed at a low level throughout the PA in WT embryos and *Hey1* was expressed more broadly in the PA (Fig 7J–7X). Expression was quantified and we found that levels of all three genes were significantly reduced in null mutant embryos (Fig 7Y). We also examined expression patterns of additional genes that are expressed in the PE.

*Isl1* (*Islet1*) is expressed in the second heart field mesoderm and endoderm, among other tissues during early embryogenesis. Based upon WMISH, there was not a dramatic difference in expression in WT, *Foxi3*^*-/-*^ and *Tbx1*^*-/-*^ embryos at E9.5 (S7A–S7C Fig). FGF signaling has been shown to act genetically downstream of *Tbx1* [48, 49] and *Foxi3* [27]. We previously found that *Fgf3* is reduced in expression when *Tbx1* is inactivated [50]. Using WMISH, we also found that *Fgf3* expression was reduced in *Foxi3*^*-/-*^ embryos at E9.5 (S7D and S7E Fig). Expression in the otic vesicle was gone because the structure doesn’t form in *Foxi3* null mutant embryos. Expression of another PE specific transcription factor gene, *Pax8* (Paired box 8) was reduced within the PA in both *Tbx1* and *Foxi3* null mutants at E9.5 (S7F–S7H Fig). *Pax8* is important for thyroid gland development and acts downstream of *Foxi3* [51]. *Pax9* is a transcription factor that is normally expressed within the PE and marks the pouches during embryogenesis [27]. We also confirmed that *Pax9* mRNA expression is reduced but not absent in the PA in *Tbx1* mutant embryos [52] in comparison to WT controls using WMISH and RNAscope (S7I–S7M Fig). Other studies reported that *Pax9* expression was mis-regulated in *Foxi3*^*-/-*^ embryos [27]. Our data indicated that *Pax9* expression was reduced in *Foxi3*^*-/-*^ embryos, although this does not rule out that it was also mis-regulated (S7J and S7M Fig). All together this shows that there are genes that act downstream of both *Tbx1* and *Foxi3*.

### Extracellular matrix proteins in *Tbx1* and *Foxi3* null mutant mouse embryos

**Fig 8:**
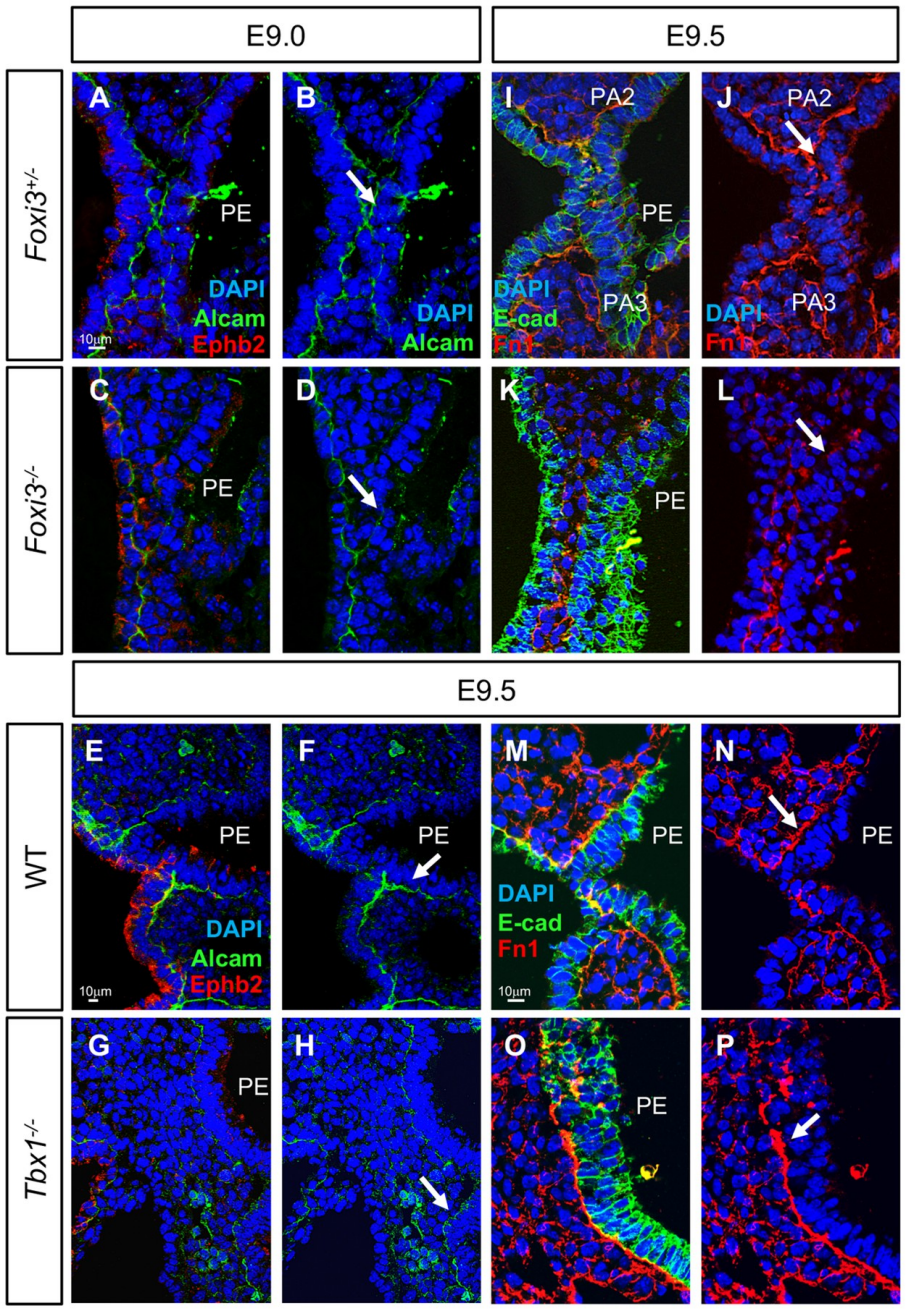
Alcam and Fibronectin expression is reduced in epithelial cells within the PA at E9.5 in *Foxi3*^*-/-*^, but not in *Tbx1*^*-/-*^ embryos. (A-H) DAPI (blue), Alcam (green) and Ephrin b2 (red) antibodies were used to examine coronal sections in embryos at E9.0. Alcam localizes to the basal side of epithelium and Ephrin b2 localizes to the apical side. Sections from *Foxi3*^*+/-*^ control embryos are shown with all three fluorescence color channels (A) and only DAPI and Alcam (B). Sections from *Foxi3*^*-/-*^ embryos are shown with all three channels (C) and only DAPI and Alcam (D). Sections from WT littermates of *Tbx1*^*-/-*^ embryos are shown with all three channels (E) and only DAPI and Alcam (F). *Tbx1*^*-/-*^ embryos are shown with all three channels (G) and only DAPI and Alcam (H). White arrows indicate where Alcam expression is reduced in *Foxi3*^*-/-*^ mutant embryos, but is present in *Tbx1*^*-/-*^ embryos in comparison to control embryos. (I-P); DAPI (blue), E-cadherin (E-cad, green) and Fibronectin (Fn1, red) antibodies mark epithelial cells and the intracellular matrix on coronal sections at E9.5. (I-J) Sections from *Foxi3*^*+/-*^ control embryos shown with all three fluorescent channels (I) and only the red and blue channels (J). (K-L) Sections from *Foxi3*^*-/-*^ embryos showing all three channels (K) and only the red and blue channels (L). White arrows indicate where expression is spotty and inconsistent in *Foxi3*^*-/-*^ mutants. (M-P) Sections from WT (M-N) and *Tbx1*^*-/-*^ embryos (O-P) showing all three channels (M and O) and only the red and blue channels (N and P). White arrow in N and P indicates where Fibronectin expression is increased in *Tbx1*^*-/-*^ mutants. n = 3 for each genotype in each experiment.

In addition to observing expression of known genes important for pharyngeal segmentation, we also investigated expression of activated leukocyte cell adhesion molecule (Alcam; also called CD166, Neurolin, or DM-GRASP), Ephrinb2, and Fibronectin (Fn1) in *Foxi3* and *Tbx1* null mutant embryos. This is because these extracellular proteins have roles in epithelial cell function and endodermal pouch formation in zebrafish [53] [54–57]. In *Foxi3*^*-/-*^ embryos, Alcam expression was reduced in the pharyngeal endoderm (Fig 8C and 8D) as compared to heterozygous controls (Fig 8A and 8B). In contrast, Alcam protein expression appeared unchanged in *Tbx1*^*-/-*^ embryos (Fig 8G and 8H) in comparison to WT controls (Fig 8E and 8F). In zebrafish, ephrinb2 is required to prevent epithelial cells from rearranging once PE segmentation is complete [58]. We did not observe a change of Ephrinb2 expression in epithelial cells in *Foxi3*^*+/-*^ and WT controls as well as *Foxi3*^*-/-*^ or *Tbx1*^*-/-*^ mutant embryos (Fig 8A, 8C, 8E and 8G). Fibronectin protein is an extracellular matrix protein that is present outside the basal surface of the epithelia closest to the adjacent mesenchyme and is also expressed in the mesenchyme [56, 57]. Fibronectin expression was absent or expression was spotty in cells adjacent to the endoderm and ectoderm in *Foxi3*^*-/-*^ mutant (Fig 8K and 8L) versus *Foxi3*^*+/-*^ control embryos (Fig 8I and 8J). Fibronectin expression in *Tbx1*^*-/-*^ mutant embryos was increased (Fig 8O and 8P) as compared to control littermates (Fig 8M and 8N), which is consistent with previous findings [59]. This indicates some differences between *Tbx1* and *Foxi3* functions. We note that some of the changes in patterns of expression could be due to morphological defects in the null mutant embryos.

## Discussion

In this report, we found that there is a genetic interaction between *Tbx1* and *Foxi3* in the formation of the thymus and parathyroid glands from the third pharyngeal pouch in mammals. Inactivation of *Foxi3* in the *Tbx1* domain resulted in absent thymus and parathyroid glands and inactivation in the endoderm resulted additionally in aortic arch defects that are similar as is observed in patients with 22q11.2DS. Expression of *Jag1*, *Hey1 Fgf3*, *Pax8* and *Pax9* was reduced in both *Tbx1* and *Foxi3* null mutant embryos, suggesting some shared downstream genes. We investigated the cellular mechanisms by which pharyngeal pouch-cleft junctions form in the process of pharyngeal segmentation. We found that the epithelial cells invaginate and form temporary multilayers in which cells become juxtaposed, repositioned and tightly intercalated to form junctions between the endoderm and ectoderm. Global inactivation of both genes resulted in failed invagination and excessive multilayers of endoderm cells. We identified autonomous and non-autonomous functions in this process. Together, this study adds new genetic, molecular and cellular insights into the process of pharyngeal segmentation in mammals.

### Epithelial cells undergo dynamic transitions in the vertebrate PA

**Fig 9:**
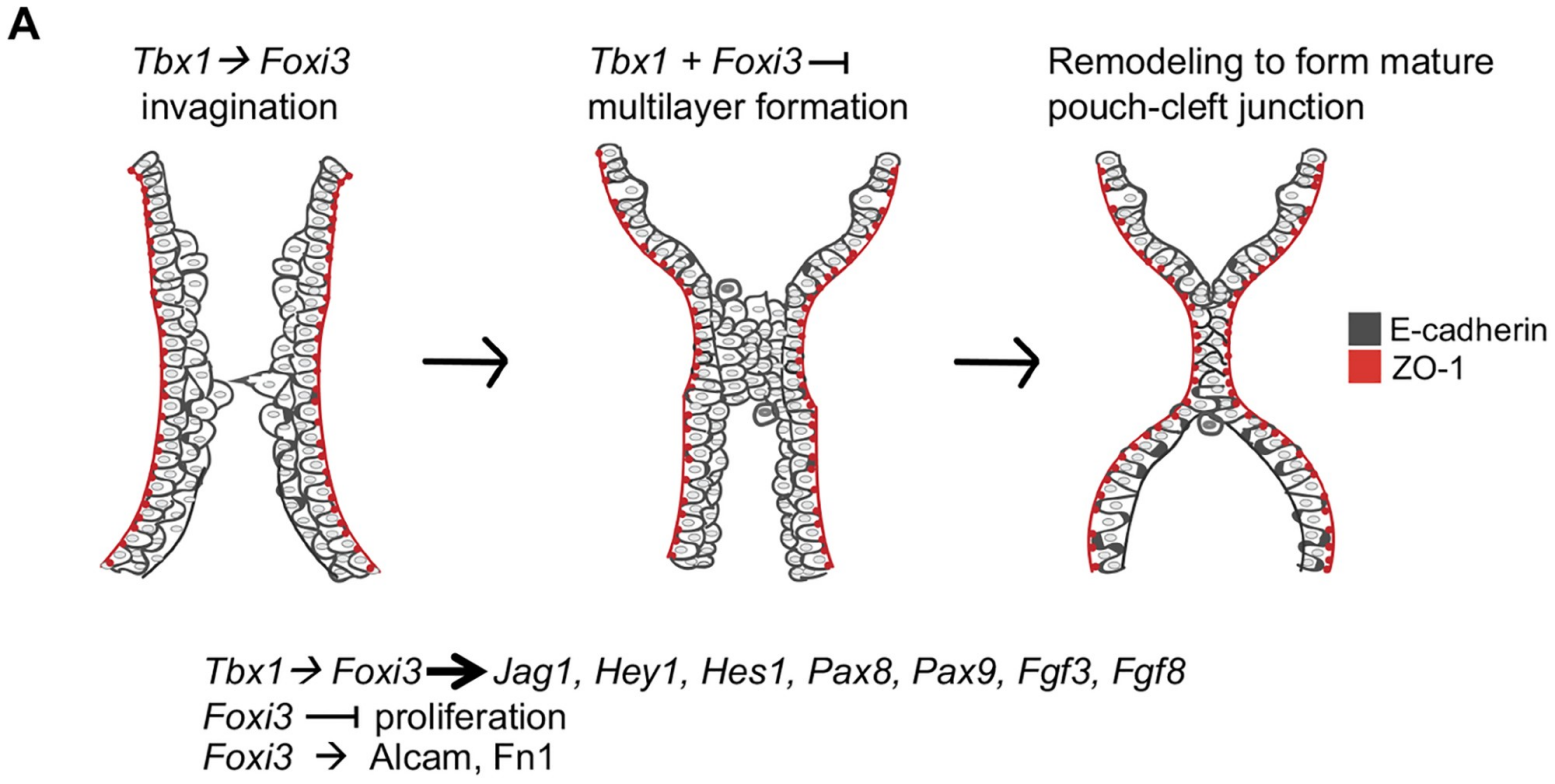
Summary cartoon of *Tbx1* and *Foxi3* functions in PA segmentation. Cartoon of epithelial cells during PA segmentation for each arch. *Tbx1* and *Foxi3* are co-expressed where segmentation to individual pharyngeal arches occurs. When segmentation of the distal PA begins at E8.5, invagination of E-cadherin expressing cells (dark gray) is initiated and a partially stratified multilayer of epithelium forms by E8.75-E9.0, near the point where invagination continues. *Tbx1* acts upstream of *Foxi3* to promote proper invagination as indicated. The outermost cell layer maintains apical/basal polarity as depicted by ZO-1 expression (red), while the inner layers do not. The epithelial cells begin to project towards each other as shown. Next, a multilayer is juxtaposed where invagination has advanced, and a zippering process ensues from the center of the region, where cells are reorganized. Finally, a dual-layer of intercalated epithelial cells are formed at the mature pouch-cleft junction and both arches are separated. *Foxi3* appears to inhibit excess proliferation of PE cells early, while *Tbx1* doesn’t alter proliferation. In *Tbx1* null mutant embryos more cells are present in the shortened PA. Inactivation of *Tbx1* or *Foxi3* results in the appearance of excessive layers of endoderm cells, in particular, where invagination is initiating. Therefore, these genes might both promote invagination and restrict excessive multilayers during PA segmentation. We found that *Tbx1* and *Foxi3* may act in the same pathway upstream of some Notch pathway genes, *Pax8* and *Pax9*, as well as *Fgf3*. Some of these genes might be required pharyngeal segmentation. It is previously known that *Tbx1* and *Foxi3* act upstream of *Fgf8*. While loss of *Foxi3* resulted in reduction of Alcam and Fibronectin expression in the extracellular matrix, global loss of *Tbx1* did not have the same role. Thus, this data explains, in part, the basis of the genetic interaction between the two genes.

During vertebrate embryonic development, the segmentation of the distal PA is needed to create individual arches that later form derivative structures including the thymus and parathyroid glands [23, 60]. We revisited the process of pharyngeal segmentation to better understand the functions of *Tbx1* and *Foxi3*. Our data indicates that there are a few major epithelial transitions required for morphogenesis, as shown in the model in Fig 9. In the first transition, invagination of the endoderm and ectoderm takes place. Next, a few layers of a partially stratified epithelium forms in the region where invagination occurs starting at E8.75-E9.0. The internal layers of epithelial cells in the partially stratified epithelium do not express the cell polarity protein, ZO-1. Interestingly, it appears as if endoderm and ectoderm cells extend processes towards each other as illustrated in Fig 9. In the final transition, as invagination is completed and the endoderm and ectoderm meet, the multilayers of loosely organized cells form a tightly organized dual intercalated pouch-cleft junction, in which ZO-1 is expressed on the apical surfaces. It can be hypothesized that a zippering process initiates in the center of the forming junction of the epithelia, in which cells become reorganized. E-cadherin expression remains throughout the process indicating that the cells retain at least some of their epithelial properties.

A similar process has been described for branching morphogenesis to form pancreatic ducts from the distal foregut endoderm [61]. In pancreatic organogenesis, a single layer of polarized foregut endoderm will invaginate into the mesenchyme to form branches and ducts composed of differentiated cells. In this process, a single layer of polarized epithelial cells is dynamically transformed to a multilayered epithelium, followed by a second transition back to a monolayer of polarized epithelial cells in the newly formed duct [61, 62]. As for the pharyngeal endoderm, the pancreatic endoderm cells express E-cadherin during this process and the row of cells on the apical side expresses ZO-1 [61, 62].

A few years ago, dynamic transitions of the pharyngeal endoderm were described in zebrafish [54]. During pharyngeal pouch formation, there is a two-step transition to form a temporary stratified epithelium from two layers of cells, which revert back to two opposing layers when pouch formation is complete [54]. In zebrafish and in mouse embryos, E-cadherin is expressed throughout the segmentation process. Apical/basal polarity is lost as detected by lack of ZO-1 expression in internal epithelial cells in zebrafish [54, 63] as we found in mouse embryos. In zebrafish, this process is regulated by signals from the ectoderm and mesoderm to the PE, and is in part, non-autonomous. Specifically, in zebrafish it was found that non-canonical Wnt (Wnt11r) signaling emanating from the mesoderm is required to regulate the process of segmentation [54]. Independently, Wnt4a signals from the ectoderm upstream of the extracellular matrix protein, Alcama (Alcam in mammals) and E-cadherin within cells, are needed to transition from multilayers to two cell layers [54].

### *Tbx1* functions to promote invagination and restrict excessive multilayer formation in the PE

*Tbx1* is expressed in the mesoderm as well as the epithelia. In zebrafish, mesodermal *tbx1* regulates expression of *wnt11r* (non-canonical Wnt) and *fgf8a* (Fibroblast growth factor 8a) morphogens that signal to the PE to promote pouch formation [20]. Similarly, in mice, inactivation of *Tbx1* in the mesoderm results failed segmentation of the distal PA [34]. Thus, the data in zebrafish and mouse is consistent for non-autonomous roles of mesodermal *Tbx1* in pharyngeal segmentation. It was previously found that *Tbx1* has autonomous roles in the PE for segmentation of the distal PA using the *Sox17*^*2A-iCre/+*^ allele [18]. We also found that invagination failed when *Tbx1* was inactivated using this allele. In *Tbx1* null mutant embryos, excessive multilayers formed by E9.5. We did not observe excessive multilayers when *Tbx1* was inactivated in the endoderm at E9.5, although there were some additional layers by E10.5. We speculate that excessive multilayer formation may be partially suppressed in endodermal conditional mutant embryos because *Tbx1* is still expressed in the mesoderm.

### Distinct roles of *Foxi3* in epithelial cell dynamics

In mammals and zebrafish, there are three *Foxi* class genes, *Foxi1*, *Foxi2* and *Foxi3*. In zebrafish, *foxi1* has a similar expression pattern and function to that of *Foxi3* [51]. Recently, it was found that inactivation of *foxi1* resulted in a failure to transition from a multilayered epithelium to a simple dual layered pharyngeal pouch and excessive multilayers formed [64]. This is similar to our findings for *Foxi3* function in mammals, however we found that *Foxi3* is also required in the invagination process. Further, in zebrafish, *foxi1* appears to have its major role in the ectoderm to signal non-autonomously to the PE [64]. In *foxi1* null mutant zebrafish, Alcama expression was normal. In contrast, in the mouse, loss of *Foxi3* in the PE resulted in failed segmentation along with reduced Alcam expression. It is possible that ectodermal *Foxi3* might have important signaling roles, but these would be independent to PE functions. Nevertheless, it appears that there are some differences in the function of these homologs in different vertebrates. There are also some differences in zebrafish in regards to *tbx1* function. In zebrafish, the *tbx1* gene does not have an autonomous role in the PE in pouch formation [20]. In contrast, *Tbx1* has autonomous roles in the PE in the mouse.

Differences in Alcam expression levels in *Foxi3* and *Tbx1* null mutant embryos, implicate some mechanistic differences in gene function on the extracellular milieu. Eph-ephrin signaling in adjacent cells is important for cell migration. In zebrafish, EphB2 and EphB3 are required to maintain E-cadherin expression during budding morphogenesis of the endoderm from the foregut [62]. During pharyngeal pouch morphogenesis in zebrafish, EphrinB signaling is required to increase E-cadherin expression in the second transition of pharyngeal segmentation [58]. In *foxi1* mutant zebrafish, expression of EphrinB2a was not changed [64]. Ephrin2a is expressed in a similar pattern to ZO-1. As in zebrafish, we found that Ephrin b2 was not reduced in *Foxi3* mutant mouse embryos, and therefore is not directly implicated downstream of *Foxi3*.

In zebrafish, *foxi1* in the pharyngeal ectoderm initiates *wnt4a* signaling and that this is required for the second transition of the endoderm to form a final mature segment [64]. In endoderm specific *Foxi3* conditional mutant mouse embryos, the ectoderm was able to invaginate properly, but segmentation failed. It is possible that endodermal cells might not have been able to properly respond to signals emanating from the ectoderm. Our data also indicates that invagination of the ectoderm is not dependent on invagination of the endoderm. Rather, the ectoderm can initiate invagination independently. This is consistent with studies performed in shark and chick embryos where the endoderm remains separate from the ectoderm throughout epithelial cell invagination [23]. Further work will need to be done to understand the role of extracellular matrix proteins and signaling on epithelial cell dynamics in mouse models.

### Shared genetic mechanisms downstream of *Tbx1* and *Foxi3*

The FGF signaling pathway was previously found to be disrupted in the pharyngeal epithelia of both *Tbx1* and *Foxi3* null mutant embryos. *Fgf8* [49] is expressed in the pharyngeal epithelia as well as the mesoderm and is required for early zebrafish [65] and mouse embryogenesis [66–68]. Inactivation of *Fgf8* in the pharyngeal epithelia in mouse embryos resulted in similar phenotypic defects in the distal pharyngeal apparatus as the *Tbx1* null mutant embryos [69]. Previous published work shows that *Fgf8* expression is reduced in the pharyngeal pouches in *Tbx1* null mutant mouse embryos and the two genes, *Tbx1* and *Fgf8* genetically interact [48]. Both *fgf3* and *fgf8* are required for segmentation of the distal PA in zebrafish [7]. Relevant to this report, *Fgf8* expression was reduced *Foxi3* null mutant mouse embryos and addition of exogenous *fgf3* partially rescued defects in *foxi1* morphants [27]. As expected, in this report, we found that *Fgf3* is reduced in expression in *Foxi3* mutant embryos. This suggests that *Tbx1* and *Foxi3* might act in the same genetic pathway as *Fgf3* and *Fgf8* as well as other FGF ligand genes. We found *Pax8* and *Pax9* expression was also reduced in the PE in *Foxi3* and *Tbx1* null mutant embryos, and it is possible that in particular, *Pax9* is critical for PA segmentation [70]. In addition, we found genes in the Notch pathway reduced in expression in both *Tbx1* and *Foxi3* null mutant embryos as well.

The Notch pathway has many diverse roles in embryogenesis by regulating Notch effectors of the Hey/Her/Hes class of transcription factors. We previously noted that *Jagged1* (*Jag1*), encoding a cell surface Notch ligand, was reduced in expression the pharyngeal arch epithelia in *Tbx1* null mutant embryos [46]. In another report, expression of *Hes1*, encoding a Notch downstream effector, was reduced in expression in *Tbx1* null mutant embryos and further, *Hes1* null mutant embryos had similar thymus and aortic arch artery defects as *Tbx1* null mutant embryos [21]. More recently, it was reported that Notch pathway genes were altered downstream of failed pharyngeal segmentation in mouse embryos due to inactivation of the transcription co-activator, *Eya1* in mouse models [71]. In that study, *Jag1*, *Hes1* and *Hey1* expression was altered or reduced in the pharyngeal epithelia [71].

In this report, we found that *Jag1*, *Hes1* and *Hey1* were reduced in expression in *Tbx1* and *Foxi3* null mutant mouse embryos. *Tbx1* is still expressed in the abnormal pharyngeal epithelia in *Foxi3* null embryos, suggesting that perhaps the loss of expression that is observed might be due to downregulation of expression of *Notch* pathway genes and other genes described above. It is possible that some of the defects that were observed could be due to reduction of Notch signaling. Although our data support a possible role, it is not known if Notch pathway genes are required for segmentation of the PA. Therefore, more studies are needed to be done in the future to test a possible role for Notch signaling in this process.

### Translational insights

Patients with 22q11.2DS have defects within structures derived from the PA including craniofacial dysmorphism, T-cell deficiencies or dysfunction, hypocalcemia, as well as aortic arch and cardiac outflow tract defects [72]. *TBX1* is the major candidate gene for these defects, and it is required for PA segmentation [17, 18, 20]. Based on results presented in this report, we suggest that *Tbx1* may act upstream of *Foxi3* in this process. One question is whether individuals might be identified that have mutations in *FOXI3*. There has been one report of a patient with a deletion of one allele of *FOXI3* that had severe ear defects, mild craniofacial defects, and missing arteries derived from PA1 and PA2 [73]. These symptoms are due to defects of structures derived from the PA but are different from those typically observed in patients with 22q11.2DS.

The phenotypic expression of 22q11.2DS varies extensively, implicating the existence of genetic or environmental modifiers. It would be interesting to determine whether DNA sequence variants in *FOXI3* or other downstream genes, such as *FGF* pathway genes, *PAX9*, or Notch pathway genes, might act as potential modifiers of phenotype in individuals with 22q11.2DS. Analysis of sequence from a large cohort of individuals with 22q11.2DS will be required to test this possibility.

## Materials and methods

### Ethics statement

All experiments using mice were carried out according to regulatory standards defined by the National Institutes of Health and the Institute for Animal Studies, Albert Einstein College of Medicine (https://www.einstein.yu.edu/administration/animal-studies/), IACUC protocol # 2016–0507.

### Mouse mutant alleles

The following mouse mutant alleles used in this study have been previously described: *Foxi3*^*f/f*^ (flox = f), *Foxi3*^*+/-*^ [27], *Tbx1*^*Cre/+*^ [36] *Sox17*^*2A-iCre/+*^ [38], *Tbx1*^*+/-*^ [11] and *ROSA26*^*GFPf/+*^ (RCE: loxP) [74]. *Foxi3*^*-/-*^ embryos were generated by inter-crossing *Foxi3*^*+/-*^ mice. Double *Tbx1* and *Foxi3* heterozygous embryos were generated by inter-crossing *Tbx1*^*+/-*^ and *Foxi3*^*+/-*^ mice. *Tbx1*^*Cre/+*^*;Foxi3*^*f/f*^ and *Sox17*^*2A-iCre/+*^*;Foxi3*^*f/f*^ embryos were generated by crossing male *Tbx1*^*Cre/+*^*;Foxi3*^*f/+*^ or *Sox17*^*2A-iCre/+*^*;Foxi3*^*f/+*^ mice with *Foxi3*^*f/f*^ females. *Foxi3*^*+/-*^, *Tbx1*^*+/-*^, *Tbx1*^*Cre/+*^*;Foxi3*^*f/+*^, *Sox17*^*2A-iCre/+*^*;Foxi3*^*f/+*^, *Sox17*^*2A-iCre/+*^*;Tbx1*^*f/+*^ and wildtype littermates were used as controls for the experiments, as indicated.

The *Foxi3*^*+/-*^, *Sox17*^*2A-iCre/+*^, and *Tbx1*^*Cre/+*^ mice were backcrossed 10 generations to a Swiss Webster background from a mixed C57Bl/6, Swiss Webster background. The PCR strategies for mouse genotyping have been described in the original reports and are available upon request.

### Mouse embryo heart histology and phenotypic analysis

Mouse embryos were isolated in phosphate-buffered saline (PBS) and fixed overnight in 10% neutral buffered formalin (Sigma Corp.). Following fixation, the embryos were dehydrated through a graded ethanol series, embedded in paraffin and sectioned at 10 μm. All histological sections were stained with hematoxylin and eosin (H&E) using standard protocols in the Einstein Histopathology Core Facility (http://www.einstein.yu.edu/histopathology/page.aspx). A total of 80 embryos, including controls, at E15.5 were obtained from more than 10 independent crosses and analyzed morphologically using light microscopy. Fisher’s exact test was used to determine if parathyroid and thymus gland defects were significant in *Tbx1*^*+/-*^*; Foxi3*^*+/-*^ compared to *Tbx1*^*+/-*^ embryos.

### RNAscope *in situ* hybridization and quantification

RNAscope *in situ* hybridization with non-radioactive mRNA probes was performed as previously described [75]. Tissue was fixed in 4% paraformaldehyde (PFA) for 24 hours at 4°C and then cryopreserved in 30% sucrose in PBS overnight at 4°C. Embryos were embedded in OCT and cryosectioned at 10 μm thickness. RNAscope probes for *Tbx1*, *Foxi3*, *Pax9*, *Hey1*, *Hes1*, *Jag1*, *Foxn1*, and *Gcm2* were generated by Advanced Cell Diagnostics. Quantification was performed using Volocity Software (Perkin Elmer Corporation) where each nuclei and mRNA signal dot were counted. Each probe was calculated separately. The ratios of cell number to number of signal dots was calculated for each embryo (n = 3). P-values were determined using the Student’s t-test.

### Whole mount *in situ* hybridization

Whole-mount RNA *in situ* hybridization with non-radioactive probes was performed as previously described [76, 77], using PCR-based probes for *Foxi3* [24], *Tbx1* [78], *Jag1* [79], *and Hey1* [80]. The probe for *Hes1* was generated from a cDNA plasmid clone [21, 81]. Following the whole mount RNA *in situ* hybridization protocol, the embryos were fixed in 4% PFA and then dehydrated through a series of graded ethanol steps, embedded in paraffin, and sectioned at 10 μm thickness. Minimum of 2–4 embryos from 2–3 independent litters were analyzed for each experiment.

### Immunofluorescence on embryo sections

Embryos were collected at various stages: E8.5 (7–10 somite pairs), E9.0 (15–19 somite pairs), E9.5 (20–23 somite pairs) E10.0 (24–29 somite pairs), and E10.5 (30–33 somite pairs). Fixation was carried out in 4% PFA in PBS at 4°C for two hours. After fixation, tissue was washed in PBS and then cryoprotected in 30% sucrose in PBS overnight at 4°C. Embryos were embedded in OCT and cryosectioned at 10 μm. After fixation, frozen sections were obtained as described and then permeabilized in 0.5% Triton X-100 for 5 min. Blocking was performed with 5% goat serum in PBS/0.1% Triton X-100 (PBT) for 1 hour. Primary antibody was diluted in blocking solution and incubated for 1 hour. Primary antibodies used included: E-cadherin (BD Transduction laboratories 610181, 1:200 mouse), ZO-1 (Invitrogen 61–7300, 1:200 rabbit), Fibronectin (ab2413, 1:100 rabbit), Alcam (R&D BAM6561, 1:50 mouse), Ephrin b2 (ab150411, 1:200 rabbit) and GFP (ab6673 1:500 goat). Proliferation of cells was assessed by immunofluorescence using antibody anti-phospho Histone H3 (Ser10), which is a mitosis marker (06–570 Millipore). Sections were washed in PBT and incubated with a secondary antibody for 1 hour. Secondary antibodies used were Alexa Fluor 488 goat a-mouse IgG (Invitrogen A32723) at 1:500 and Alexa Fluor 568 donkey a-rabbit IgG (Invitrogen A11019) at 1:500. Slides were mounted in hard-set mounting medium with DAPI (Vector Labs H-1500). Images were then captured using a Zeiss Axio Observer microscope with an apotome.

### Cell number and proliferation quantification on tissue sections

To count epithelial cell number, we obtained 10 μm serial coronal sections of control, *Tbx1*^*-/-*^, *Foxi3*^*-/-*^, and *Sox17*^*2A-iCre/+*^*;Foxi3*^*f/f*^ embryos, which were collected and stained with an antibody for E-cadherin. To ensure that the cell quantification was accurate, we counted E-cadherin positive cells in the PA in every other section throughout each embryo. We did not count epithelial cells that were not part of the PA. When counting cells, we matched the embryos by stage using somite counts, and we matched the sections by position within the embryo. We also ensured that for each pair of control and mutant embryos, we counted the same number of sections (10–12 per embryo). We counted all phosphoH3 positive epithelial cells in each section and calculated the ratio of proliferating cells within the pharyngeal epithelium. For *Tbx1*^*-/-*^ mutant embryos, since the PA is shorter in comparison to WT littermates, we also calculated the size of the PA using ImageJ. We then counted the E-cadherin positive cells of the PA, marking the epithelium, and divided this number by the size of the PA. The mean and standard error of the average cell counts for controls and mutant embryos were determined and they were compared using the t-test. Representations of the complete PA region from at least 3–6 embryos per genotype from at least 3 independent litters were used in each assay.

## Supporting information

S1 Fig Phenotype of third arch derivatives in *Tbx1*^*+/-*^*;Foxi3*^*+/-*^ embryos. (A-F) Transverse histology sections stained with H&E of *Foxi3*^*+/-*^ (A-B), *Tbx1*^*+/-*^ (C-D) and *Foxi3*^*+/-*^*;Tbx1*^*+/-*^ (E-F) embryos at E15.5. Abbreviations: parathyroid (PT), thyroid (T) and thymus (Thy). (G-H) Tables summarizing parathyroid (G) and thymus (H) defects observed. Numbers of glands observed was indicated (two per embryo). *Foxi3*^*+/-*^, n = 8; *Tbx1*^*+/-*^, n = 13 and *Foxi3*^*+/-*^*;Tbx1*^*+/-*^, n = 14 embryos at E15.5. Normally parathyroid glands are adjacent to thyroid glands (A). Parathyroid glands were scored as being ectopic when they were more caudally located than the thyroid glands in mutant embryos (C, E). Thymus glands were noted as hypoplastic that were smaller in size (F) than normal (B, D). Ectopic thymus glands were scored as such that were more rostrally located in comparison to control embryos (F). (I-M) Coronal histology sections stained with H&E of WT (I-J) and *Tbx1*^*+/-*^*;Foxi3*^*+/-*^ (K-M) embryos at E10.5. Black arrows indicate the third pharyngeal pouch (I-M). Related to Figs 1 and 2.
(TIF)

S2 Fig *Foxi3* expression is reduced in *Tbx1*^*Cre/+*^*;Foxi3*^*f/f*^ and *Sox17*^*2A-iCre/+*^*;Foxi3*^*f/f*^ embryos. (A-H) WMISH using antisense *Foxi3* mRNA probe on *Tbx1*^*Cre/+*^*;Foxi3*^*f/+*^ control (A-B), *Tbx1*^*Cre/+*^*;Foxi3*^*f/f*^ conditional mutant embryos (C-D), *Sox17*^*2A-iCre/+*^*;Foxi3*^*f/+*^ control (E-F) and *Sox17*^*2A-iCre/+*^*;Foxi3*^*f/f*^ conditional mutant embryos (G-H) with corresponding coronal sections. Black arrows indicate where *Foxi3* mRNA expression is reduced within conditional mutant embryos. Related to Figs 1–4 and 6.
(TIF)

S3 Fig *Tbx1*^*Cre/+*^*;Foxi3*^*f/f*^ embryos have no heart or arch artery defects. (A-F) Transverse histology sections stained with H&E of WT control (A-C) and *Tbx1*^*Cre/+*^*;Foxi3*^*f/f*^ mutant (D-F) embryos. (G-H) India ink was injected into the ventricle of *Tbx1*^*Cre/+*^*;Foxi3*^*f/+*^ control (G) and mutant (H) embryos at E10.5 to visualize the aortic arches. (I-L) Transverse histology sections of *Tbx1*^*Cre/+*^*;Foxi3*^*f/-*^ mutant embryos at E15.5. Abbreviations: right subclavian artery (R. sub), thymus (Thy), aorta (Ao), pulmonary trunk (PT), right atrium (RA), left atrium (LA), right ventricle (RV), left ventricle (LV), ventricular septum (VS). Related to Figs 1 and 4.
(TIF)

S4 Fig Phenotypes in *Foxi3*^*-/-*^ and *Sox17*^*2A-iCre/+*^*;Foxi3*^*f/f*^ mutant embryos. (A-C) Whole mount images of *Foxi3*^*+/-*^ control (A), *Foxi3*^*-/-*^ (B), and *Sox17*^*2A*-*iCre/+*^*;Foxi3*^*f/f*^ (C) embryos at E9.5. Arrow in A indicates PA3. Asterisks in B, indicate the hypoplastic first arch and in C, indicate the distal PA that failed to segment to arches. (D-F) Transverse histology sections stained with H&E of *Sox17*^*2A-iCre/+*^*;Foxi3*^*f/f*^ embryos at E15.5. RRSA is indicated (D), IAAB is present and indicated by the yellow arrow (E). Abbreviations: aorta (Ao), right ventricle (RV), left ventricle (LV), and retro-esophageal right subclavian artery (RRSA). (G-L) India ink was injected into the ventricle of WT control (G, J), *Foxi3*^*-/-*^ (H, K) and *Sox17*^*2A-iCre/+*^*;Foxi3*^*f/f*^ (I, L) embryos at E10.5. The right and left side of these embryos are shown. Arrows indicate absent 4^th^ aortic arch arteries in both genotypes of mutant embryos. Related to Figs 1 and 6.
(TIF)

S5 Fig Phenotypes in *Tbx1* null and conditional null mutant embryos. (A-F) Proliferation assay on coronal sections of WT (A-C) and *Tbx1*^*-/-*^ mutant embryos (D-F) using a phospho-H3 (ph3, red) antibody to mark cells undergoing mitosis. E-cadherin antibody (green) was utilized to visualize the epithelial cells. E8.5 (A and D) and E9.5 (B-C and E-F) staged embryos were analyzed. White boxes in B and E indicate area of magnification; n = 3 for both stages and genotypes. (G) Quantification of proliferation assay. The mitotic index is the ratio of proliferating cells to total cell counts of epithelium within the PA. The t-test was used to calculate P-values as shown. (H-K) DAPI (blue), E-cadherin (green), and ZO-1 (red) antibodies were used to visualize epithelial cells within the PA. Epithelial cells in WT (H-I) *Tbx1*^*-/-*^ (J-K) coronal sections were visualized at E8.5 (H and J) and E9.5 (I and K); n = 3 each genotype. PE indicates pharyngeal endoderm. Arrows in H, indicate the position in the PA where cells are invaginating. (L) Quantification of cell numbers to proportion of the size of the PA. (M-P) *Sox17*^*2A-iCre/+*^*;Tbx1*^*f/+*^ controls and *Sox17*^*2A-iCre/+*^*;Tbx1*^*f/f*^ conditional mutant embryos at E9.5 (M-N) and E10.5 (O-P; n = 3 for both stages and genotypes). Related to Fig 5.
(TIF)

S6 Fig Epithelial cell proliferation analysis in *Foxi3* mutant embryos. (A-L) Proliferation assay was performed using a phospho-H3 (pH3) antibody on coronal sections of WT control (A-D), *Foxi3*^*-/-*^ (E-H), and *Sox17*^*2A-iCre/+*^*;Foxi3*^*f/f*^ (I-L) mutant embryos at E8.5 and E9.5. At E8.5 and E9.5; a total of n = 6 and n = 4, respectively, were analyzed for each control and mutant embryo. Related to Fig 6.
(TIF)

S7 Fig Expression of genes in *Foxi3*^*-/-*^ and *Tbx1*^*-/-*^ mutant embryos at E9.5. (A-C) WMISH was performed using an *Isl1* antisense probe on WT (A), *Foxi3*^*-/-*^ (B), and *Tbx1*^*-/-*^ (C) mutant embryos at E9.5. (D-E) WMISH was performed using an *Fgf3* probe on WT (D) and *Foxi3*^*-/-*^ (E) mutant embryos at E9.5. (F-H) WMISH was performed using a *Pax8* probe on WT (F), *Foxi3*^*-/-*^ (G) and *Tbx1*^*-/-*^ (H) mutant embryos at E9.5. (I-K) *Pax9* probe on WT control (I), *Foxi3*^*-/-*^ (J) and *Tbx1*^*-/-*^ (K) mutant embryos; n = 2–4 for each probe and genotype. (L-M) RNAscope *in situ* hybridization with an mRNA probe for *Pax9* (green) on coronal sections in WT (L) and *Foxi3*^*-/-*^ (M) embryos at E9.5; n = 2. PE indicates the pharyngeal endoderm. Related to Fig 7.
(TIF)
