# Supplementary figures and images for: Tbx1 and Foxi3 genetically interact in the pharyngeal pouch endoderm in a mouse model for 22q11.2 deletion syndrome
Source: PLoS Genet. 2019 Aug 14;15(8):e1008301. doi: 10.1371/journal.pgen.1008301 (PMC6709926; doi:10.1371/journal.pgen.1008301)

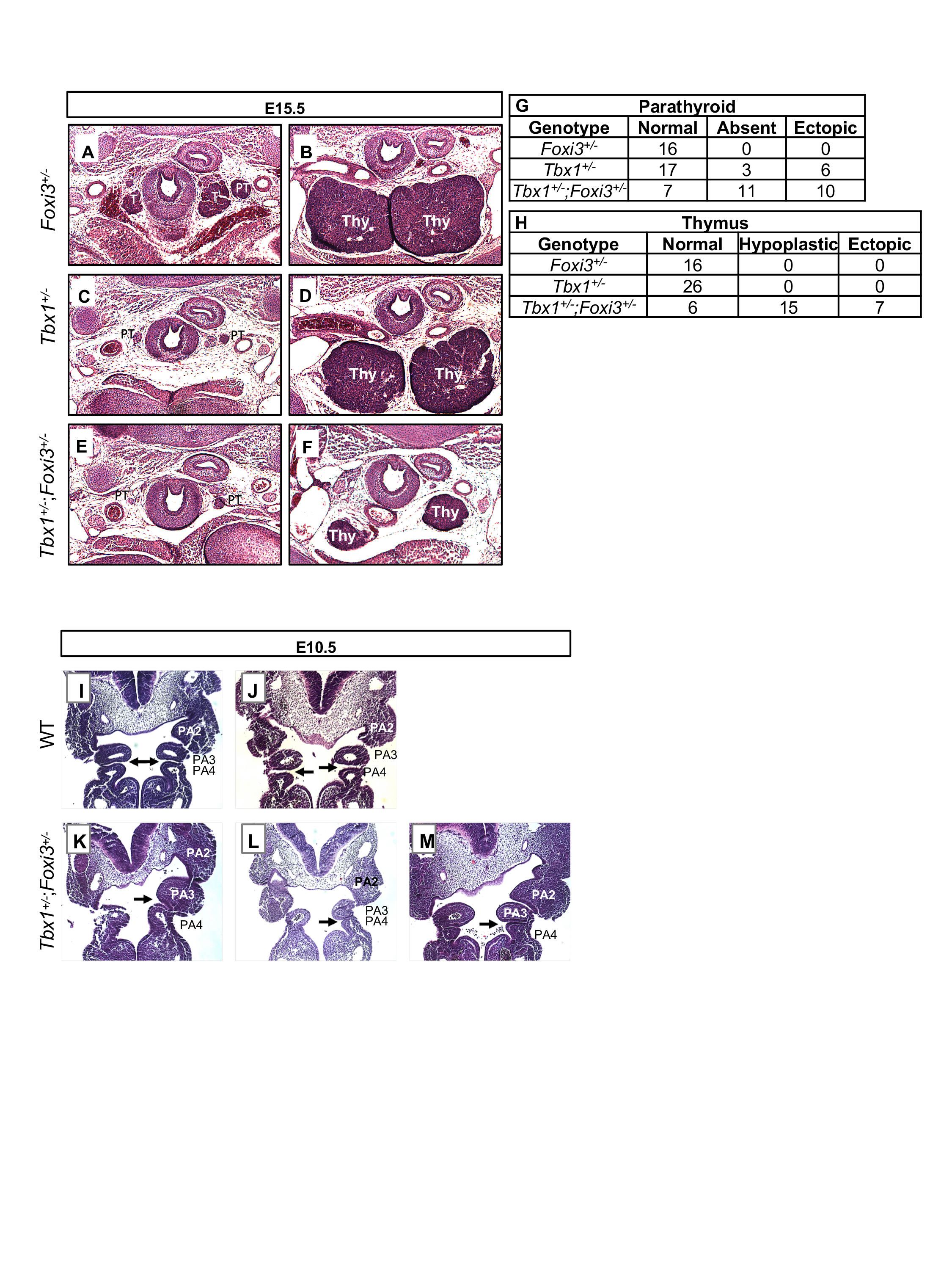

Supplement: S1 Fig — (A-F) Transverse histology sections stained with H&E of Foxi3+/- (A-B), Tbx1+/- (C-D) and Foxi3+/-;Tbx1+/- (E-F) embryos at E15.5. Abbreviations: parathyroid (PT), thyroid (T) and thymus (Thy). (G-H) Tables summarizing parathyroid (G) and thymus (H) defects observed. Numbers of glands observed was indicated (two per embryo). Foxi3+/-, n = 8; Tbx1+/-, n = 13 and Foxi3+/-;Tbx1+/-, n = 14 embryos at E15.5. Normally parathyroid glands are adjacent to thyroid glands (A). Parathyroid glands were scored as being ectopic when they were more caudally located than the thyroid glands in mutant embryos (C, E). Thymus glands were noted as hypoplastic that were smaller in size (F) than normal (B, D). Ectopic thymus glands were scored as such that were more rostrally located in comparison to control embryos (F). (I-M) Coronal histology sections stained with H&E of WT (I-J) and Tbx1+/-;Foxi3+/- (K-M) embryos at E10.5. Black arrows indicate the third pharyngeal pouch (I-M). Related to Figs 1 and 2. (TIF) [file pgen.1008301.s001.tif]

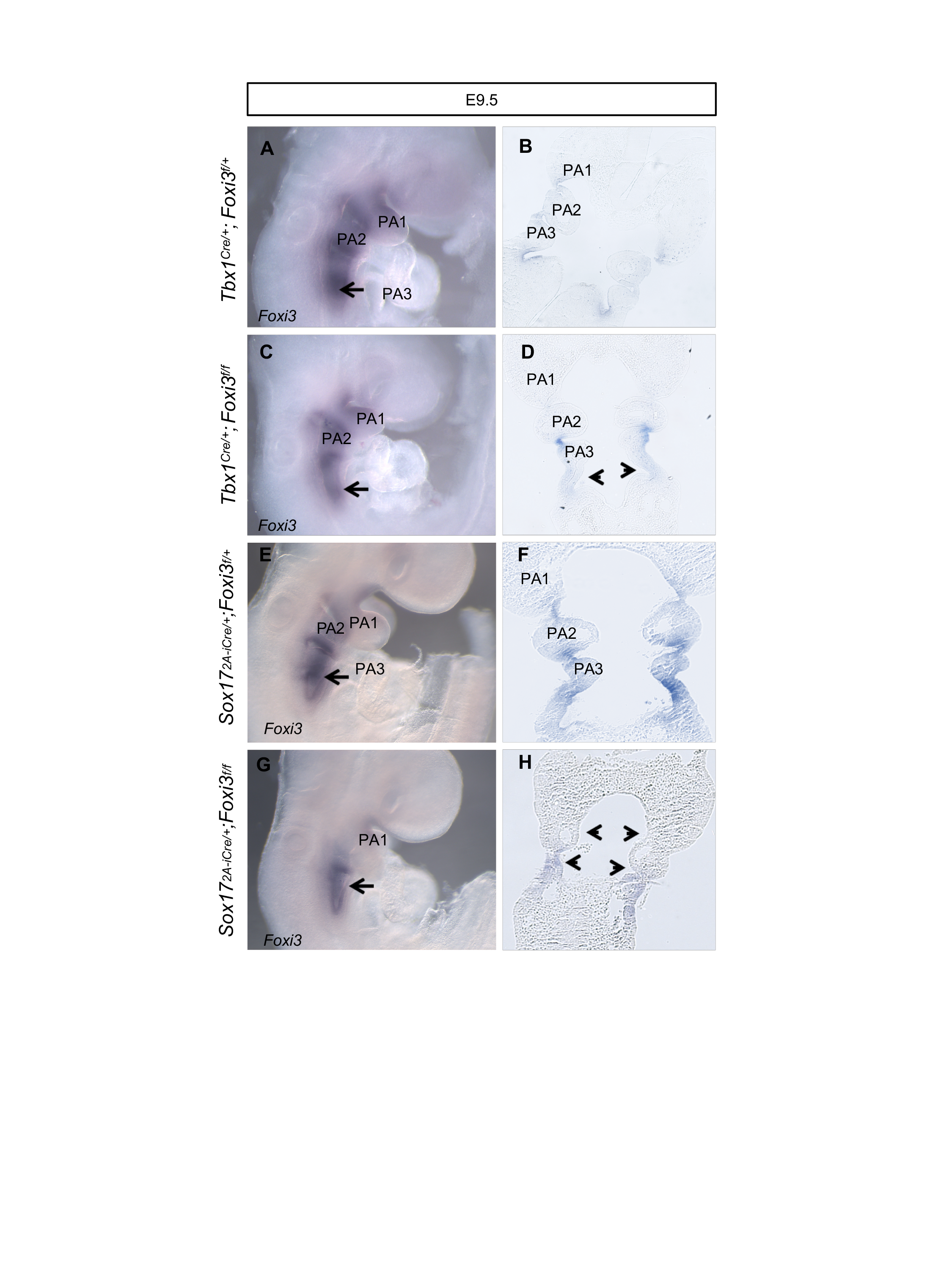

Supplement: S2 Fig — (A-H) WMISH using antisense Foxi3 mRNA probe on Tbx1Cre/+;Foxi3f/+ control (A-B), Tbx1Cre/+;Foxi3f/f conditional mutant embryos (C-D), Sox172A-iCre/+;Foxi3f/+ control (E-F) and Sox172A-iCre/+;Foxi3f/f conditional mutant embryos (G-H) with corresponding coronal sections. Black arrows indicate where Foxi3 mRNA expression is reduced within conditional mutant embryos. Related to Figs 1–4 and 6. (TIF) [file pgen.1008301.s002.tif]

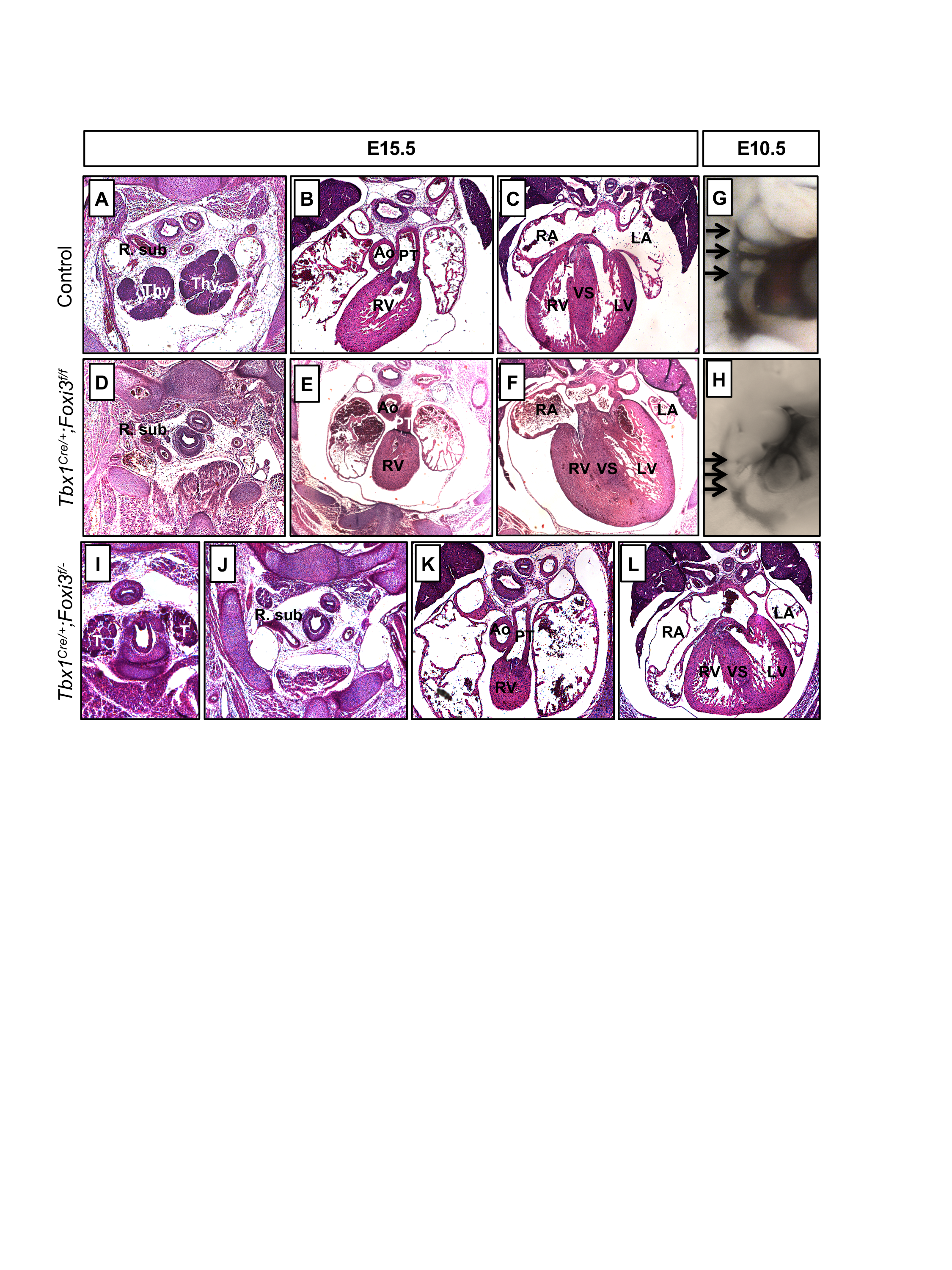

Supplement: S3 Fig — (A-F) Transverse histology sections stained with H&E of WT control (A-C) and Tbx1Cre/+;Foxi3f/f mutant (D-F) embryos. (G-H) India ink was injected into the ventricle of Tbx1Cre/+;Foxi3f/+ control (G) and mutant (H) embryos at E10.5 to visualize the aortic arches. (I-L) Transverse histology sections of Tbx1Cre/+;Foxi3f/- mutant embryos at E15.5. Abbreviations: right subclavian artery (R. sub), thymus (Thy), aorta (Ao), pulmonary trunk (PT), right atrium (RA), left atrium (LA), right ventricle (RV), left ventricle (LV), ventricular septum (VS). Related to Figs 1 and 4. (TIF) [file pgen.1008301.s003.tif]

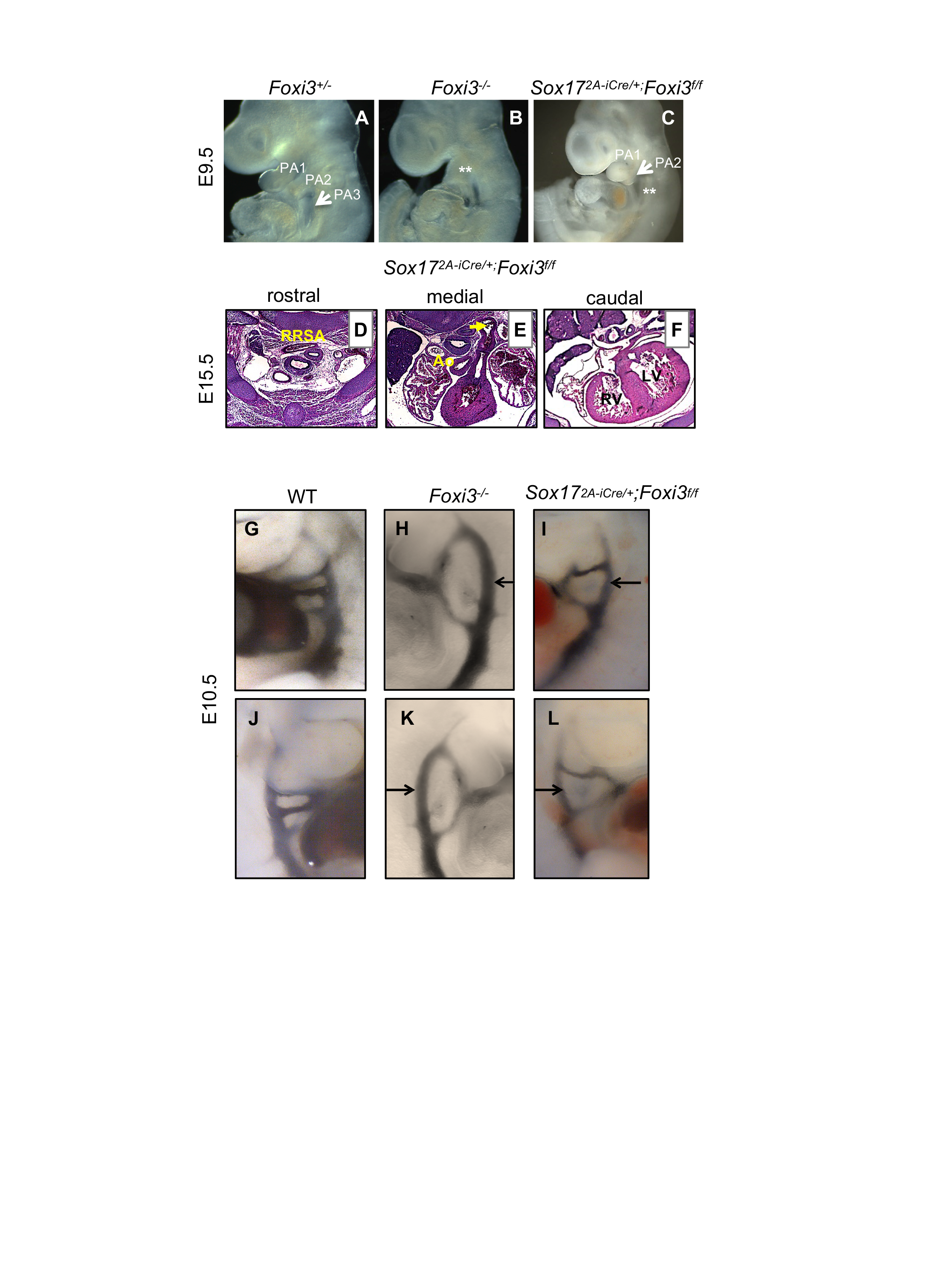

Supplement: S4 Fig — (A-C) Whole mount images of Foxi3+/- control (A), Foxi3-/- (B), and Sox172A-iCre/+;Foxi3f/f (C) embryos at E9.5. Arrow in A indicates PA3. Asterisks in B, indicate the hypoplastic first arch and in C, indicate the distal PA that failed to segment to arches. (D-F) Transverse histology sections stained with H&E of Sox172A-iCre/+;Foxi3f/f embryos at E15.5. RRSA is indicated (D), IAAB is present and indicated by the yellow arrow (E). Abbreviations: aorta (Ao), right ventricle (RV), left ventricle (LV), and retro-esophageal right subclavian artery (RRSA). (G-L) India ink was injected into the ventricle of WT control (G, J), Foxi3-/- (H, K) and Sox172A-iCre/+;Foxi3f/f (I, L) embryos at E10.5. The right and left side of these embryos are shown. Arrows indicate absent 4th aortic arch arteries in both genotypes of mutant embryos. Related to Figs 1 and 6. (TIF) [file pgen.1008301.s004.tif]

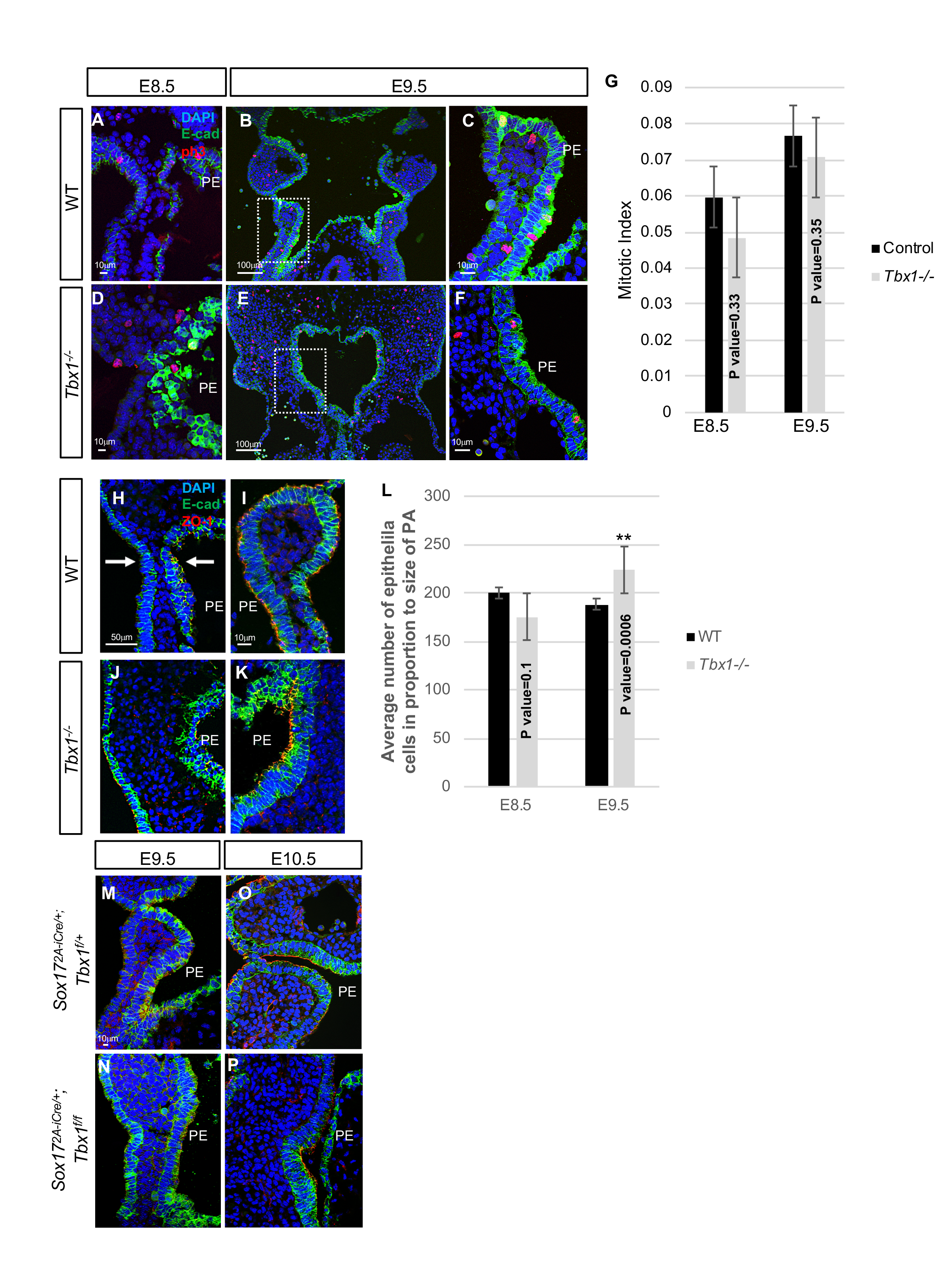

Supplement: S5 Fig — (A-F) Proliferation assay on coronal sections of WT (A-C) and Tbx1-/- mutant embryos (D-F) using a phospho-H3 (ph3, red) antibody to mark cells undergoing mitosis. E-cadherin antibody (green) was utilized to visualize the epithelial cells. E8.5 (A and D) and E9.5 (B-C and E-F) staged embryos were analyzed. White boxes in B and E indicate area of magnification; n = 3 for both stages and genotypes. (G) Quantification of proliferation assay. The mitotic index is the ratio of proliferating cells to total cell counts of epithelium within the PA. The t-test was used to calculate P-values as shown. (H-K) DAPI (blue), E-cadherin (green), and ZO-1 (red) antibodies were used to visualize epithelial cells within the PA. Epithelial cells in WT (H-I) Tbx1-/- (J-K) coronal sections were visualized at E8.5 (H and J) and E9.5 (I and K); n = 3 each genotype. PE indicates pharyngeal endoderm. Arrows in H, indicate the position in the PA where cells are invaginating. (L) Quantification of cell numbers to proportion of the size of the PA. (M-P) Sox172A-iCre/+;Tbx1f/+ controls and Sox172A-iCre/+;Tbx1f/f conditional mutant embryos at E9.5 (M-N) and E10.5 (O-P; n = 3 for both stages and genotypes). Related to Fig 5. (TIF) [file pgen.1008301.s005.tif]

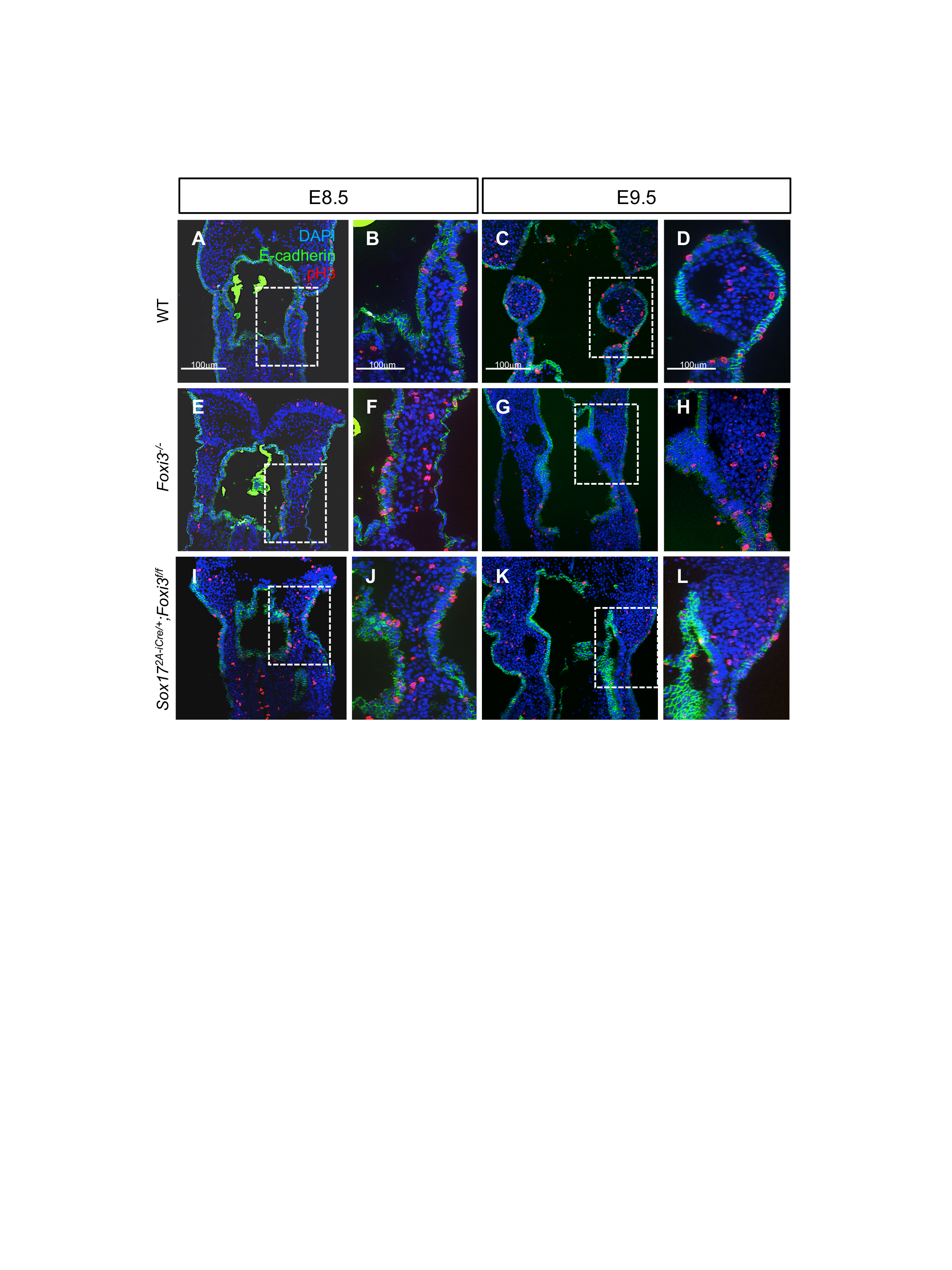

Supplement: S6 Fig — (A-L) Proliferation assay was performed using a phospho-H3 (pH3) antibody on coronal sections of WT control (A-D), Foxi3-/- (E-H), and Sox172A-iCre/+;Foxi3f/f (I-L) mutant embryos at E8.5 and E9.5. At E8.5 and E9.5; a total of n = 6 and n = 4, respectively, were analyzed for each control and mutant embryo. Related to Fig 6. (TIF) [file pgen.1008301.s006.tif]

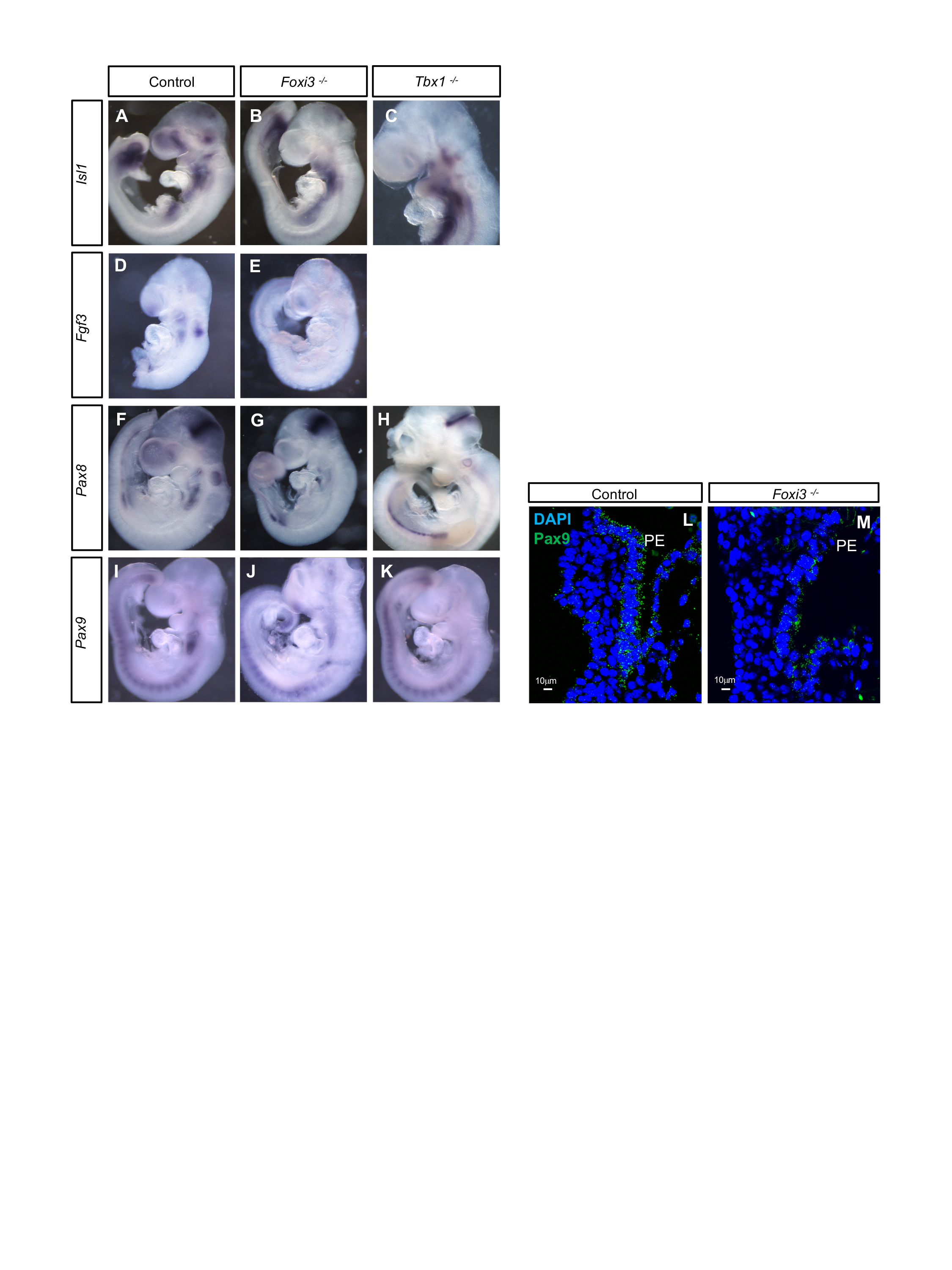

Supplement: S7 Fig — (A-C) WMISH was performed using an Isl1 antisense probe on WT (A), Foxi3-/- (B), and Tbx1-/- (C) mutant embryos at E9.5. (D-E) WMISH was performed using an Fgf3 probe on WT (D) and Foxi3-/- (E) mutant embryos at E9.5. (F-H) WMISH was performed using a Pax8 probe on WT (F), Foxi3-/- (G) and Tbx1-/- (H) mutant embryos at E9.5. (I-K) Pax9 probe on WT control (I), Foxi3-/- (J) and Tbx1-/- (K) mutant embryos; n = 2–4 for each probe and genotype. (L-M) RNAscope in situ hybridization with an mRNA probe for Pax9 (green) on coronal sections in WT (L) and Foxi3-/- (M) embryos at E9.5; n = 2. PE indicates the pharyngeal endoderm. Related to Fig 7. (TIF) [file pgen.1008301.s007.tif]
